# Supplementary material for: Cytotoxic CD8+ T cells promote granzyme B-dependent adverse post-ischemic cardiac remodeling
Source: Nat Commun. 2021 Mar 5;12:1483. doi: 10.1038/s41467-021-21737-9 (PMC7935973; doi:10.1038/s41467-021-21737-9)
Supplement: Supplementary file 1 — Supplementary Information [file 41467_2021_21737_MOESM1_ESM.pdf]

Supplementary information

Cytotoxic CD8+ T cells promote granzyme B-dependent adverse post-ischemic cardiac remodeling

Icia Santos-Zas, Jeremie Lemarié, Ivana Zlatanova, Marine Cachanado, Jean-Christophe Seghezzi, Hakim Benamer, Pascal Goube, Marie Vandestienne, Raphael Cohen, Maya Ezzo, Vincent Duval, Yujiao Zhang, Jin-Bo Su, Alain Bizé, Lucien Sambin, Philippe Bonnin, Maxime Branchereau, Christophe Heymes, Corinne Tanchot, José Vilar, Clement Delacroix, Jean-Sebastien Hulot, Clement Cochain, Patrick Bruneval, Nicolas Danchin, Alain Tedgui, Ziad Mallat, Tabassome Simon, Bijan Ghaleh, Jean-Sébastien Silvestre, Hafid Ait-Oufella

Supplementary Figures 1-38

Supplementary Tables 1-6

Supplementary Fig. 1

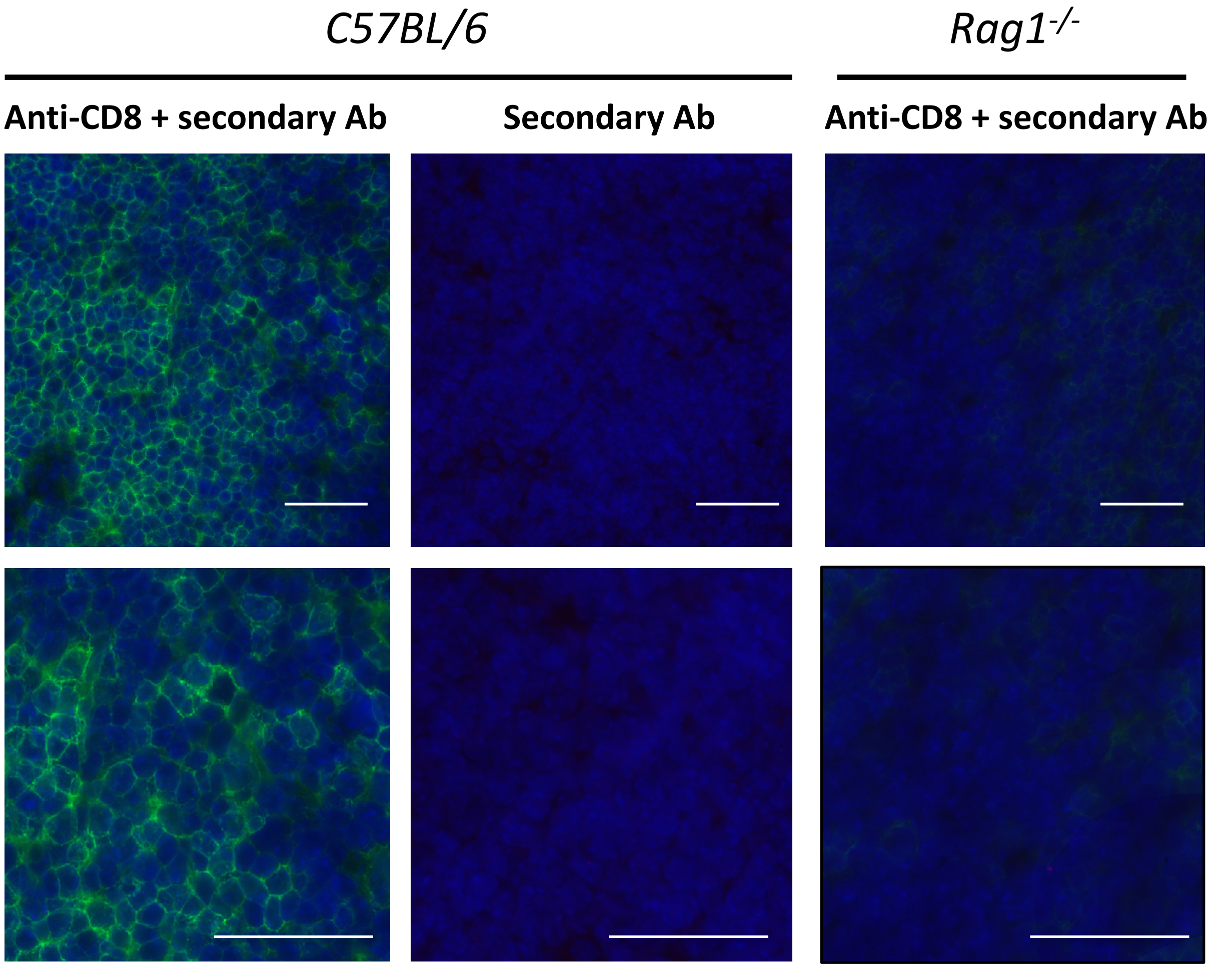

Supplementary Fig.1. Validation of anti-CD8 monoclonal antibody for immunostaining. CD8 staining was detected on spleen section of *C57Bl6* mouse but not on spleen section of lymphocyte-deficient *Rag1*$^{-/-}$ mouse. Bar scale 40μm.

Supplementary Fig. 2

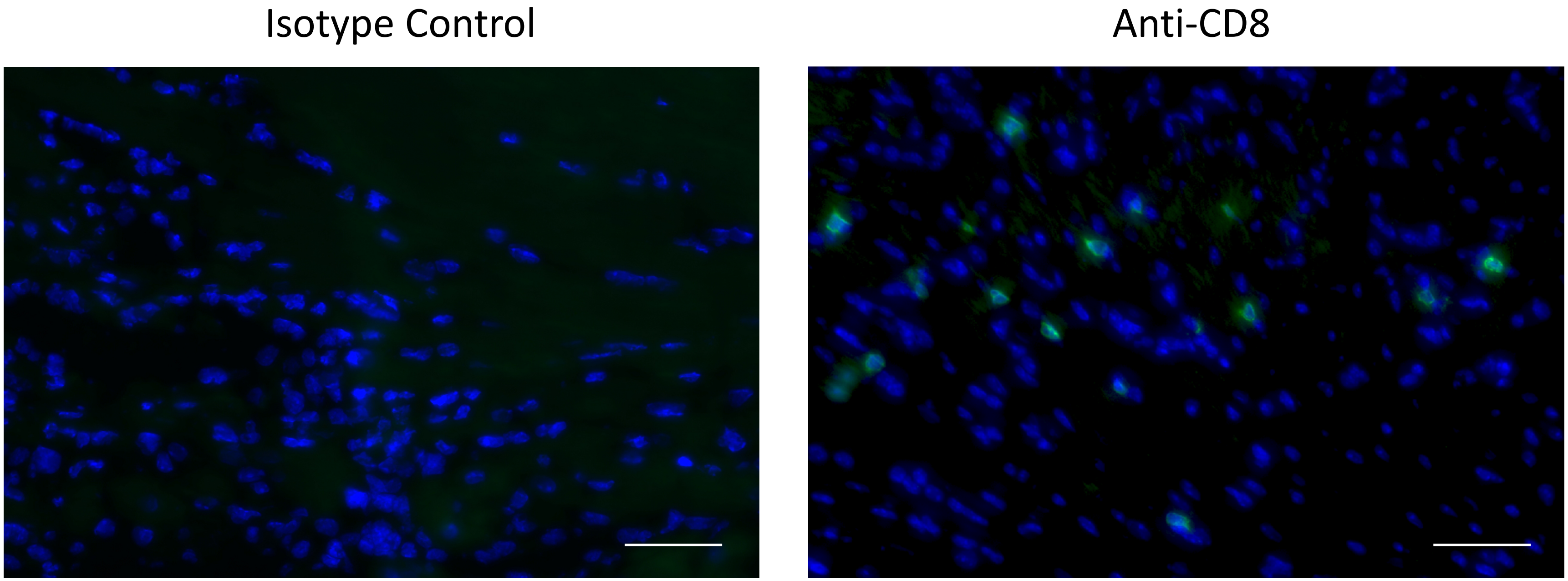

Supplementary Fig.2. Validation of anti-CD8 monoclonal antibody for immunostaining. At day 3 after MI, ischemic heart tissue sections were stained either with rat anti-mouse IgG2b isotype or with anti-CD8 monoclonal antibody. No staining was detected after isotype staining whereas CD8+ cells (Green) were detected using CD8 mAb. Scale bar 50 μm.

Supplementary Fig. 3

CTR

CD8 Depleted

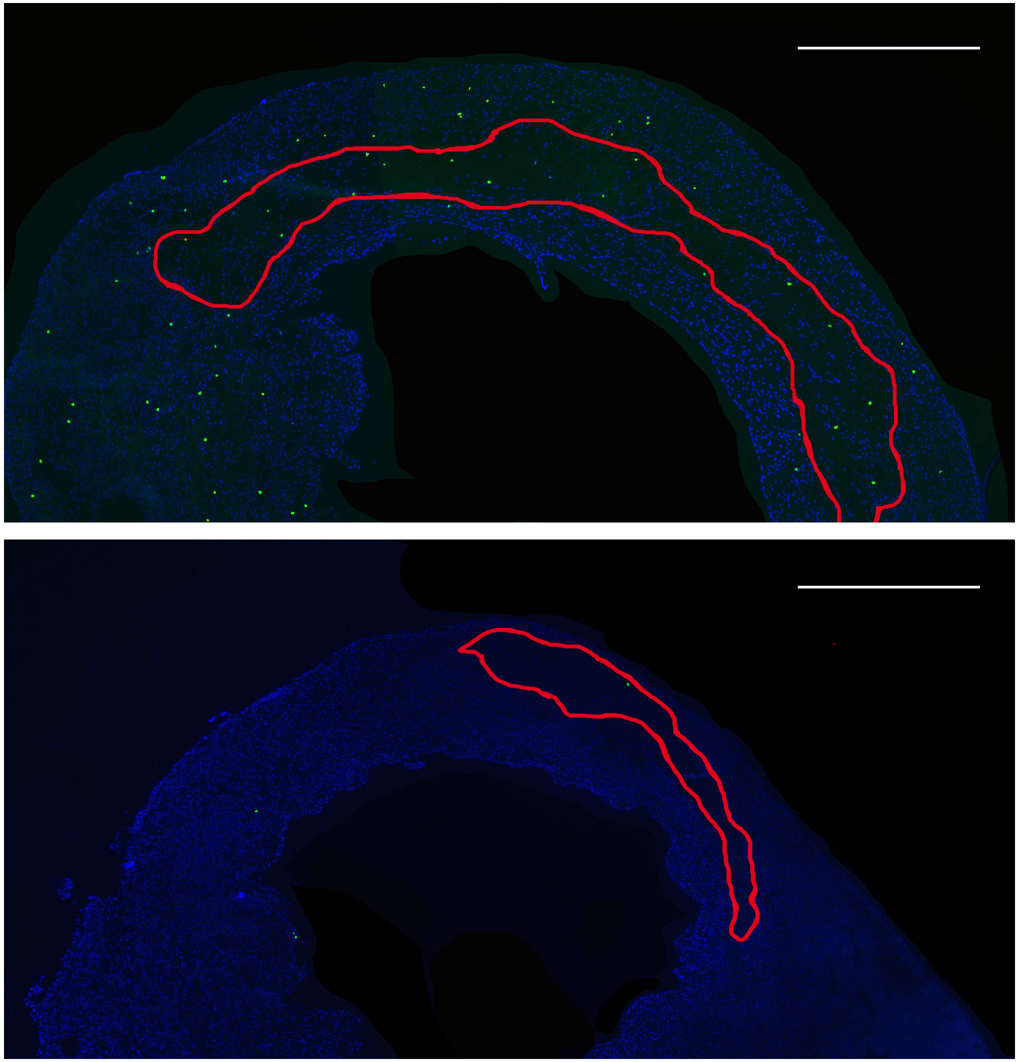

Supplementary Fig.3. Following MI, $CD8^+$ T cells infiltration was detected in both infarct and peri-infarct areas. Upper panel, immunostaining in the ischemic myocardium at day 3 after MI showing $CD8^+$ T infiltration (Green). Lower panel, CD8 immunostaining at day 3 after MI in CD8-depleted mice. Scale bar 500 μm.

Supplementary Fig. 4

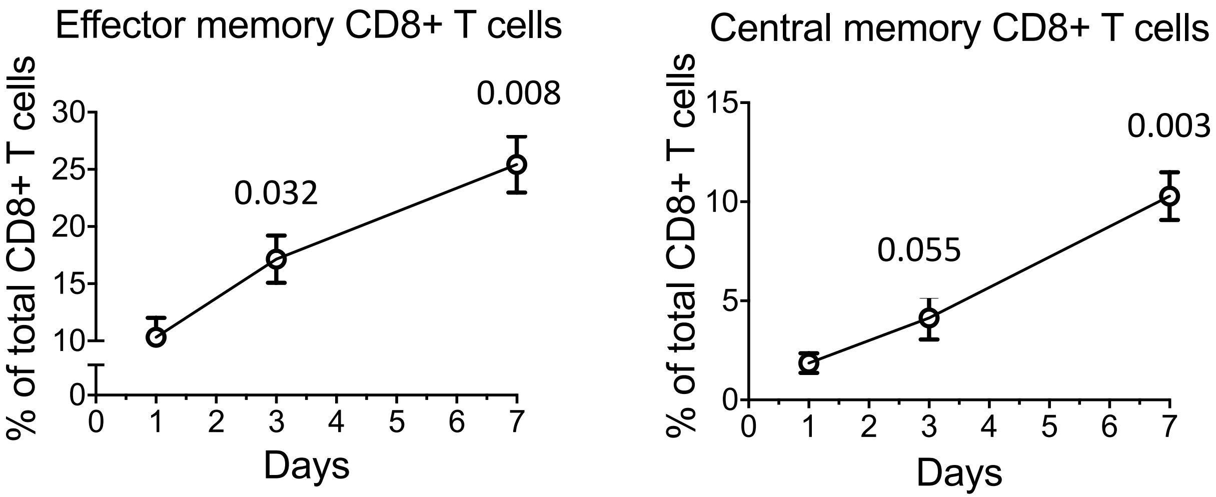

Supplementary Fig.4. Myocardial infarction induces time-dependent recruitment of effector and central memory CD8+ T cells. Proportion of $CD44^{+}CCR7^{high}$ central memory and $CD44^{+}CCR7^{low}$ effector memory $CD8^{+}$ T cells in the ischemic heart at day 1 (n=6), 3 (n=7) and 7 (n=8) after MI. mean values ± SEM are represented. P values were calculated using two-tailed Kruskal-Wallis test.

Supplementary Fig. 5

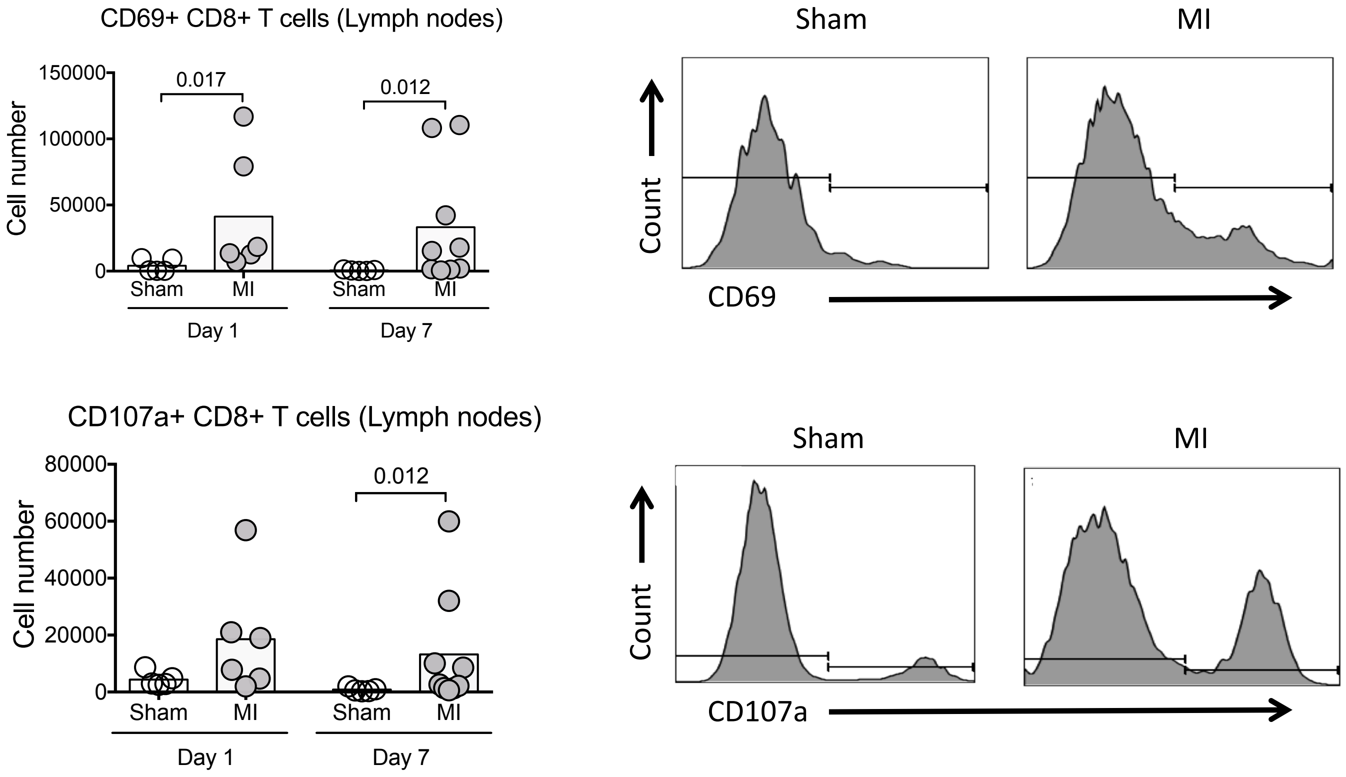

Supplementary Fig.5. Myocardial infarction induces time-dependent activation of CD8+ T cells in mediastinal lymph nodes. (a) Representative examples and quantitative analysis of CD8+ T cell expressing CD69 in mediastinal lymph nodes in MI (Grey, n=6/9 at day 1/7) and Sham operated (White, n=6/timepoint) mice. Representative example and quantitative analysis of CD8+ T cell expressing CD107a in mediastinal lymph nodes (n=6-8 mice per group/time point). P values were calculated using two-tailed Mann-Whitney test at each time point.

Supplementary Fig. 6

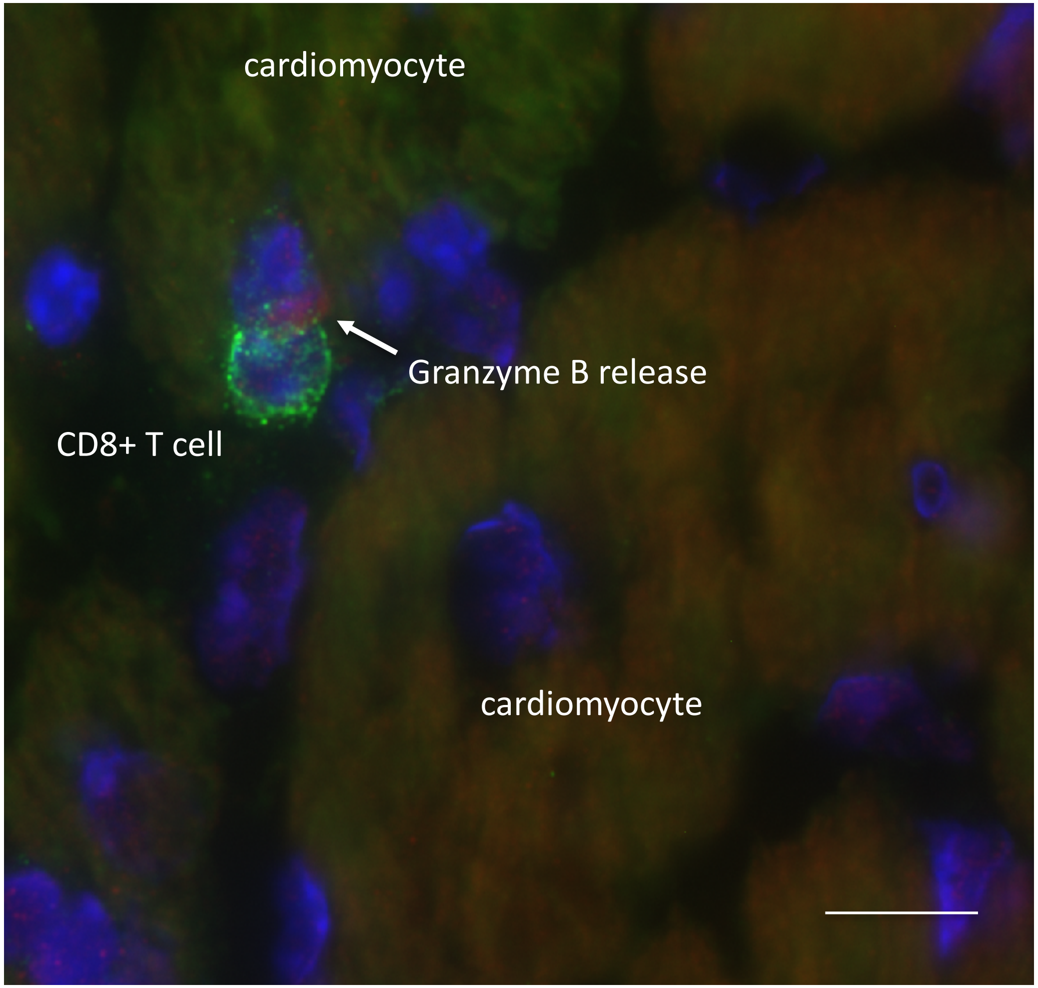

Supplementary Fig.6. CD8$^+$ T cells release Granzyme B in peri infarct area. Immunostaining in the ischemic myocardium at day 3 after MI showing CD8$^+$ T cells (Green) and Granzyme B degranulation (Red); Scale bar 20µm.

Supplementary Fig. 7

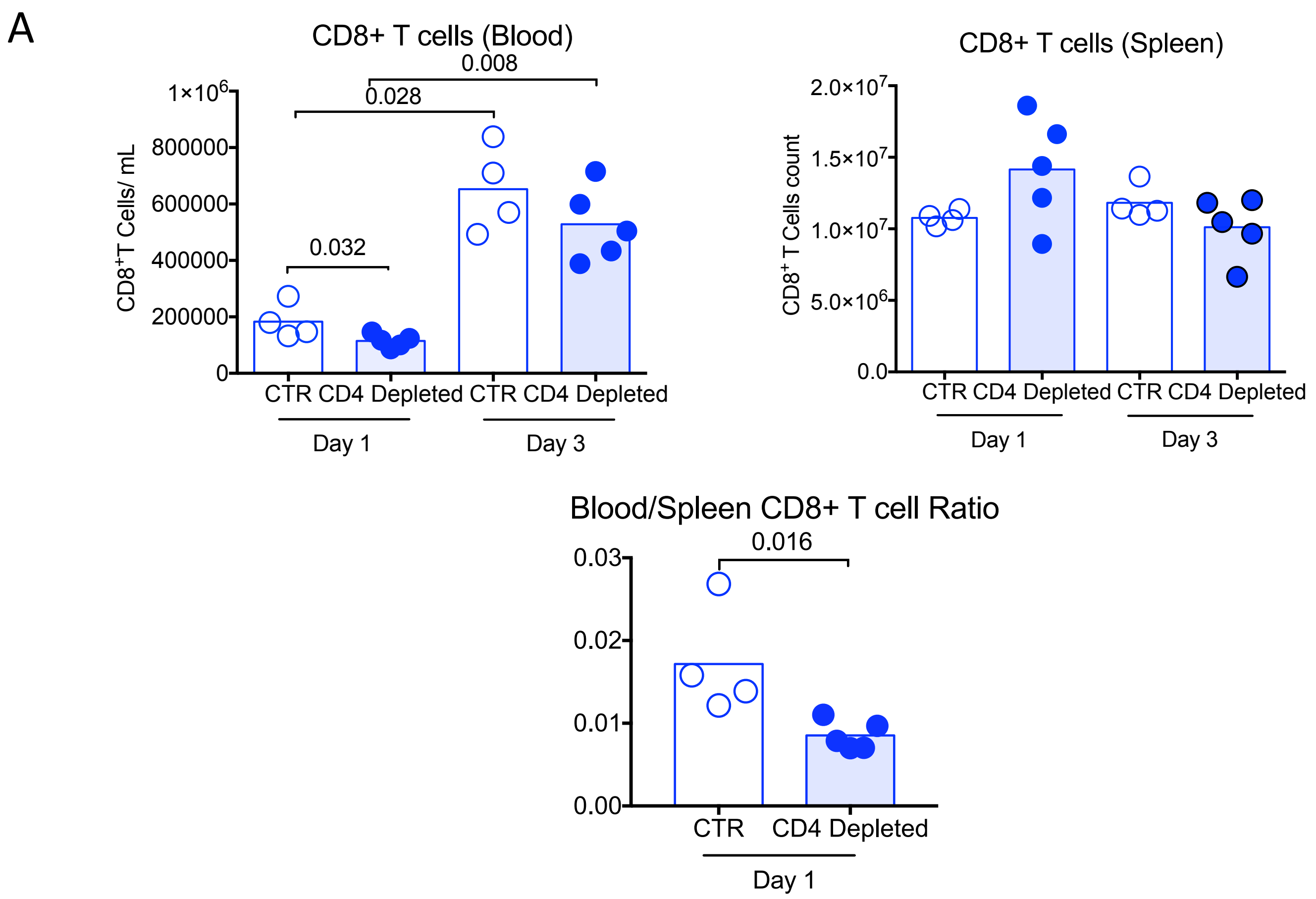

Supplementary Fig.7. CD4+ T cells regulate CD8+ T cell trafficking in the context of acute MI. *C57Bl6* Wild-type mice received intraperitoneal injection of anti-CD4 depleting monoclonal antibody (Blue filled)(150 µg/mice) or isotype control (Blue borderline) one day before coronary occlusion and CD8+ T cells were quantified in the blood (A) and in the spleen (B) at day 1 and day 3 after MI (CTR n=4/time point, CD4 Depleted n=5/time point). To evaluate the impact of CD4 depletion on CD8+ T cell mobilization from the spleen to the blood, the Blood/Spleen ratio was calculated at day 1 (C). P values were calculated using two-tailed Mann-Whitney test.

Supplementary Fig. 8

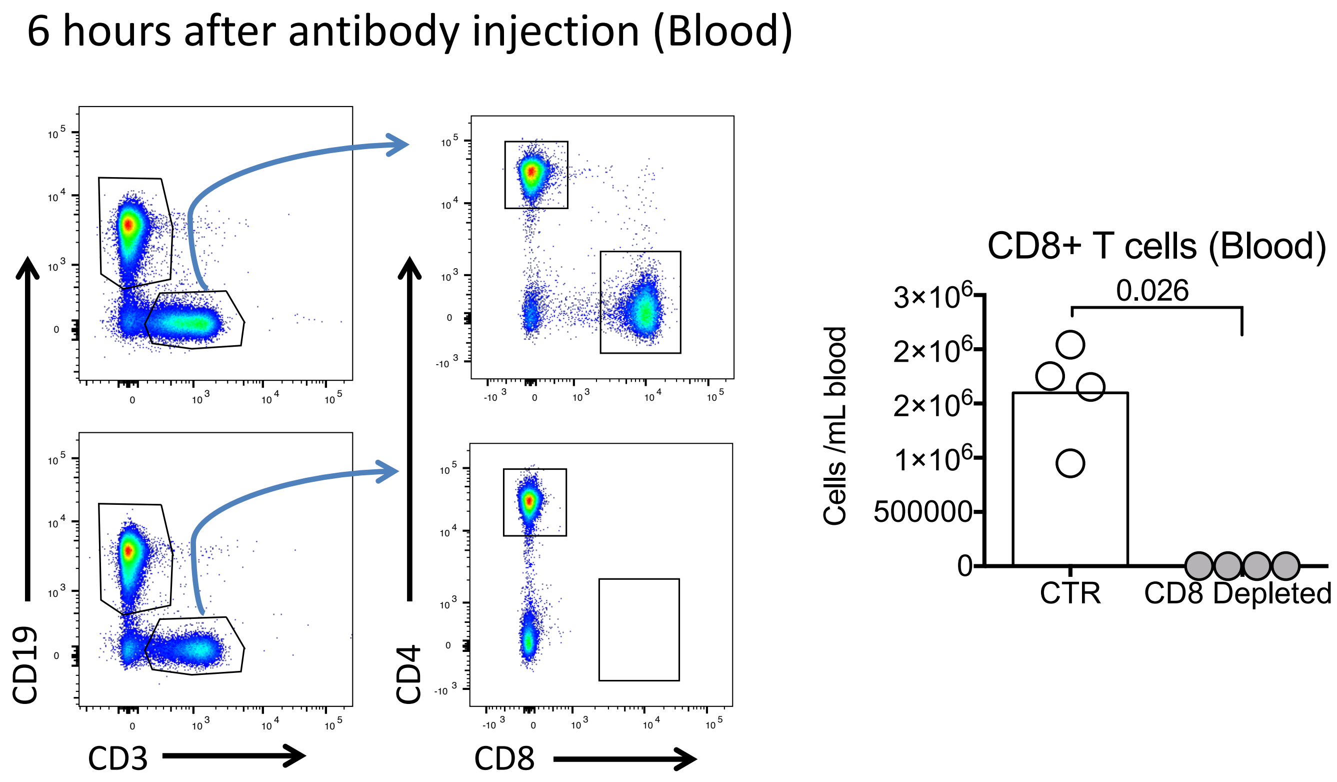

Supplementary Fig.8. mAb CD8 treatment quickly depletes circulating CD8⁺ T cells. Quantitative analysis of CD8⁺ T cell staining in the blood of C57BL/6J mice 6 hours after treatment with isotype control (CTR, white) or the CD8 mAb depleting antibody (CD8 Depleted, Grey) (n=4/Group). P values were calculated using two-tailed Mann-Whitney test.

Supplementary Fig. 9

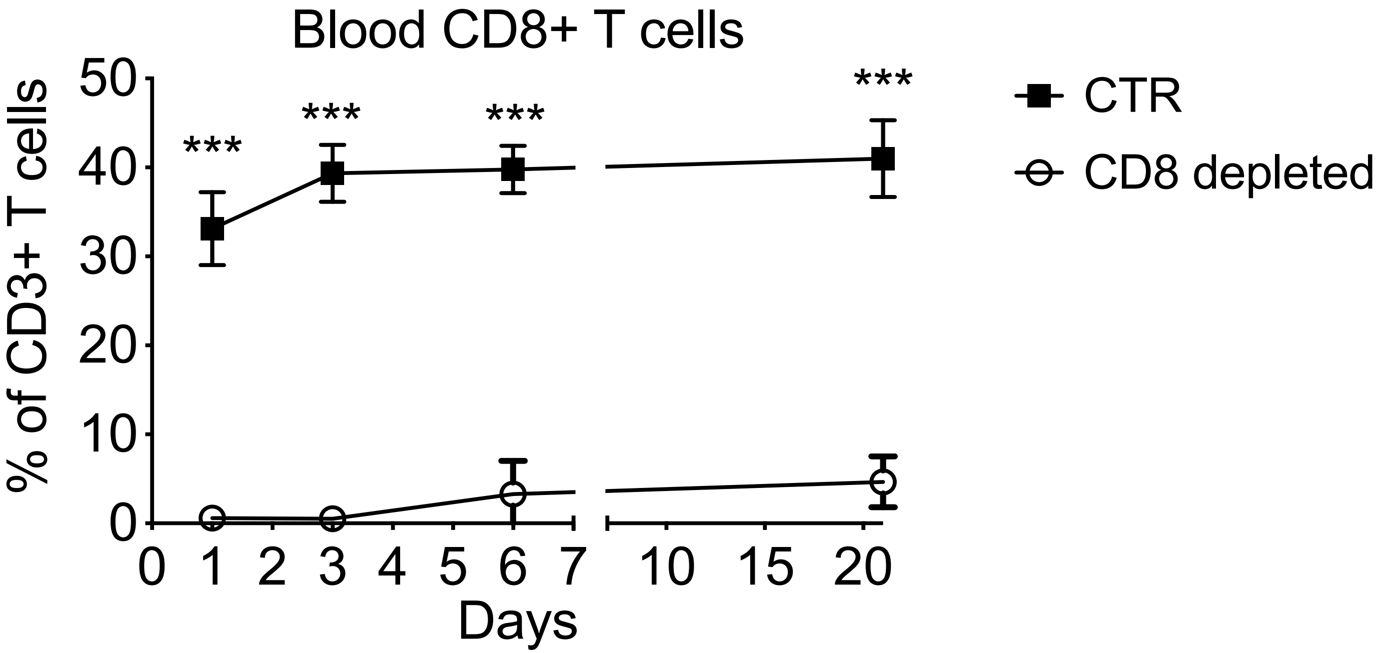

Supplementary Fig.9. mAb CD8 treatment efficiently depletes circulating CD8⁺ T cells. Quantitative analysis of CD8⁺ T cell staining in the blood of C57BL/6J mice treated with isotype control (CTR, Black box) or the CD8 mAb (CD8 Depleted, white circle) until Day 21 (n=6/group/time point); mean values ± SEM are represented, *** P<0.001. P values were calculated using two-tailed Mann-Whitney test at each time point.

Supplementary Fig. 10

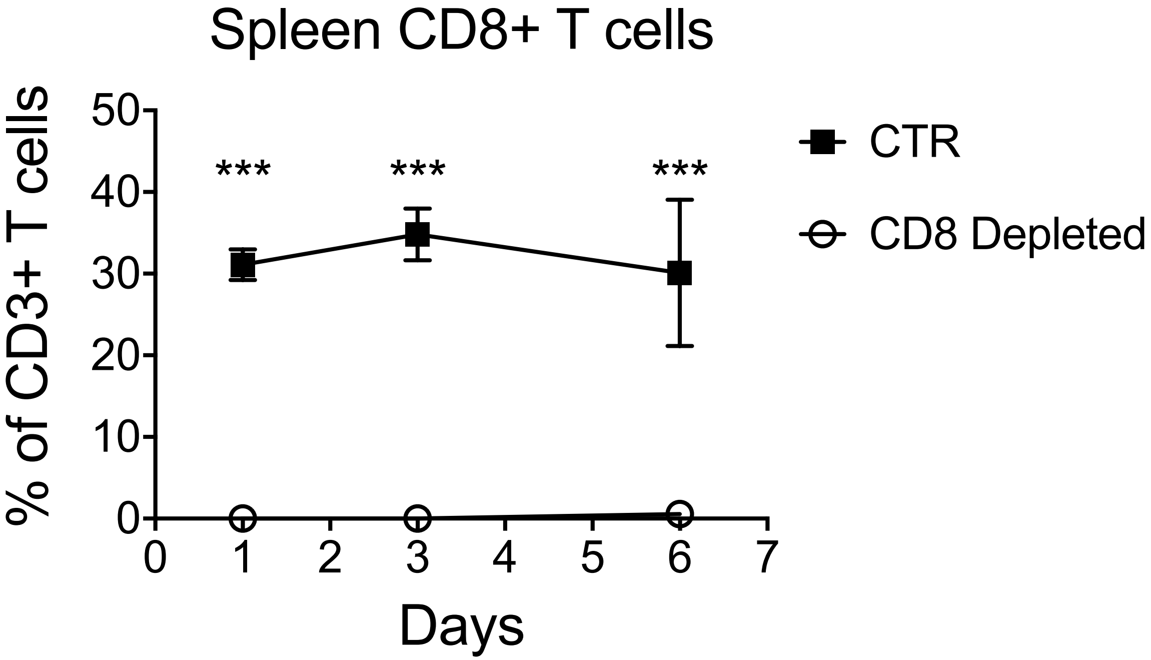

Supplementary Fig.10. mAb CD8 treatment efficiently depletes spleen CD8⁺ T cells. Quantitative analysis (right) of CD8⁺ T cell staining in the spleen of C57BL/6J mice treated with isotype control (CTR, black box) or with the CD8 mAb (CD8 Depleted, white circle) (n=6/group/time point); mean values ± SEM are represented, *** $P<0.001$. P values were calculated using two-tailed Mann-Whitney test at each timepoint.

Supplementary Fig. 11

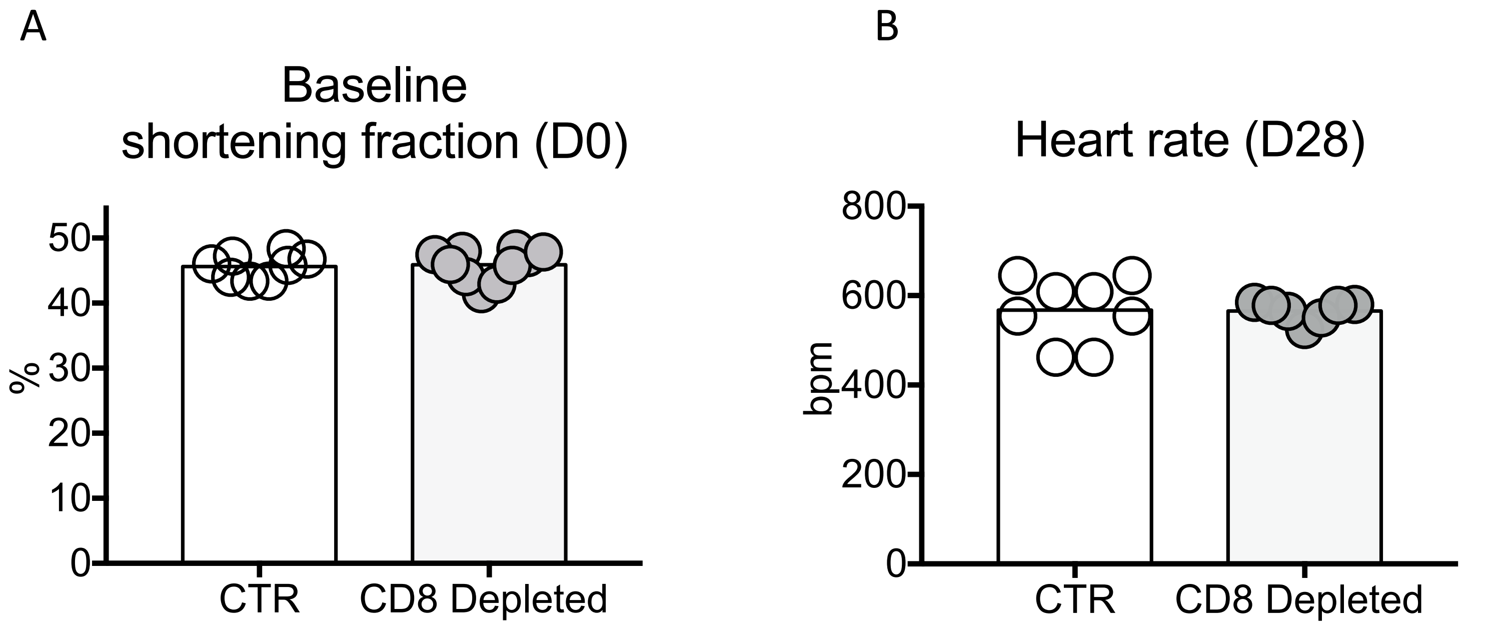

Supplementary Fig.11. (A) Baseline echocardiography-derived parameters before isotype (White circle) or CD8 monoclonal antibody treatment (Grey circle) (n=10/group) and **(B)** heart rate at day 21 after MI (CTR n=8, CD8 depleted n=9).

Supplementary Fig. 12

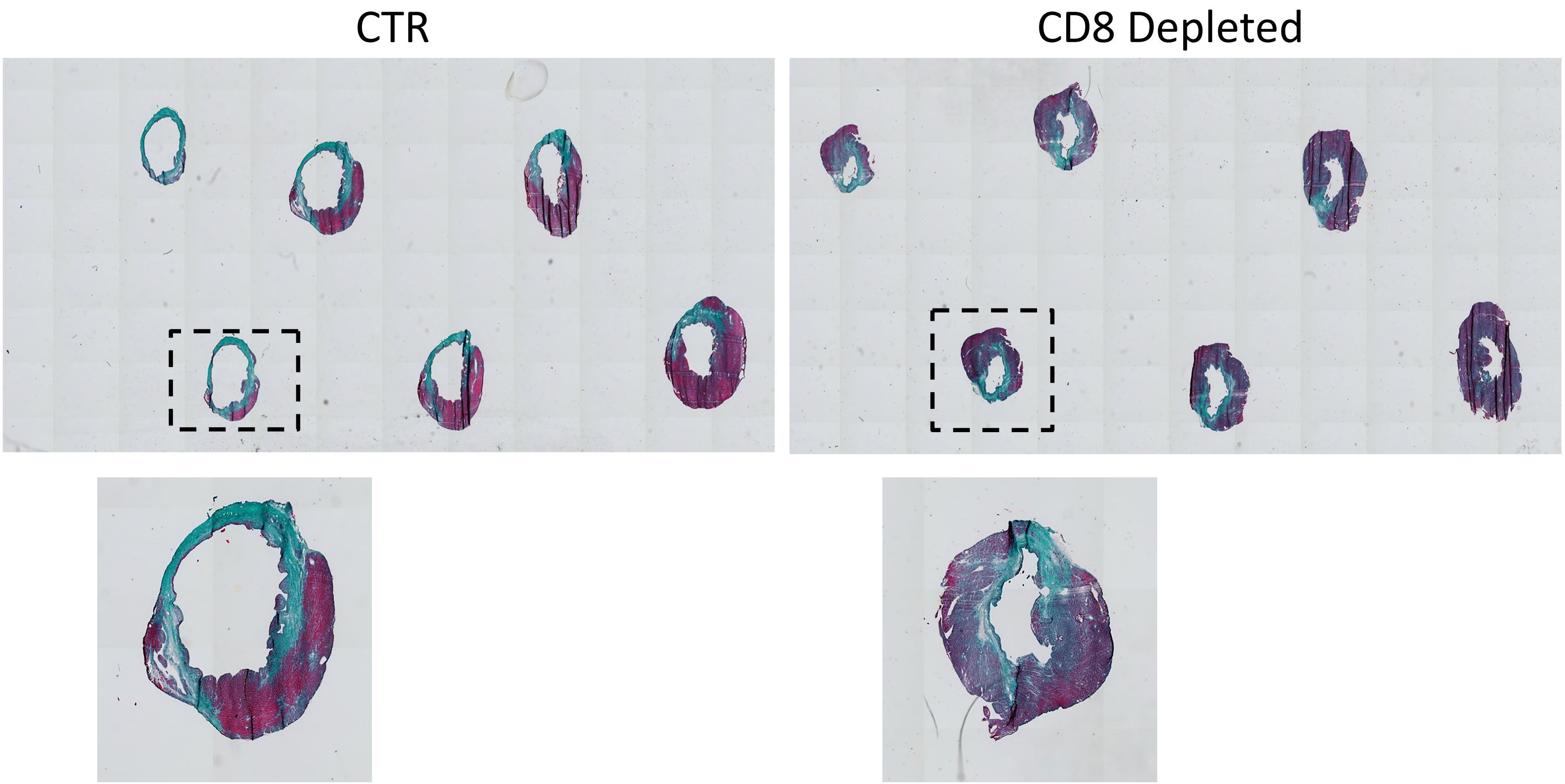

Supplementary Fig.12. mAb CD8 treatment reduces infarct size at Day 21. Representative photomicrographs of infarct size evaluation using Masson Trichrome staining on sections from the entire heart of isotype-treated or anti-CD8 depleted mice at day 21 after MI.

Supplementary Fig. 13

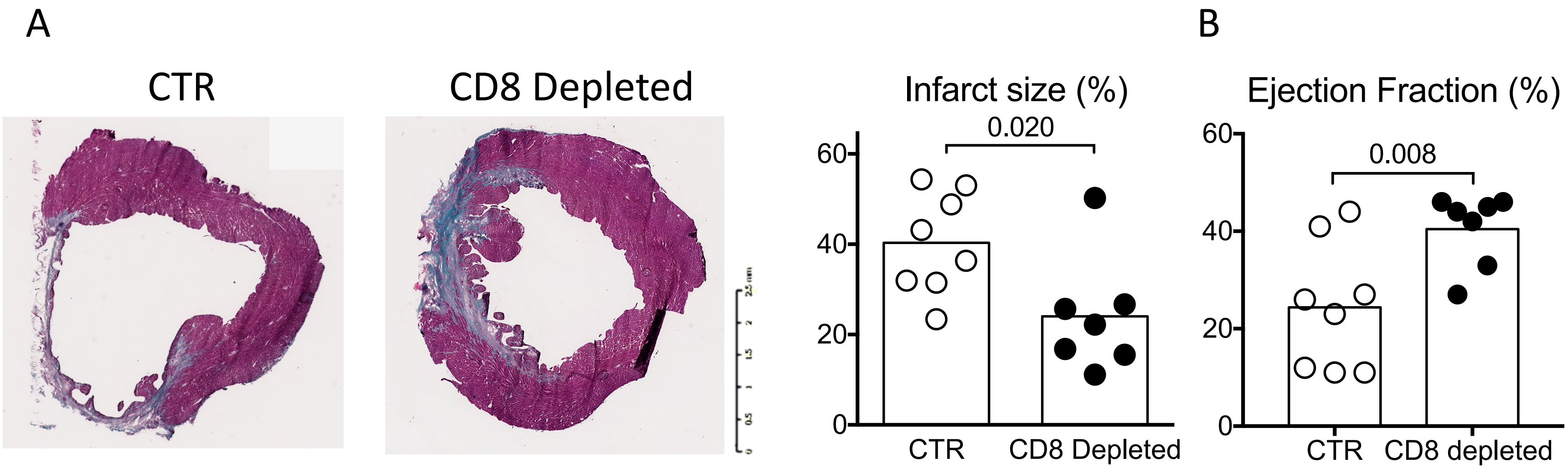

Supplementary Fig.13. mAb CD8 treatment limits deleterious post-ischemic cardiac remodeling at Day 21 in females C57BL/6 mice. A, Representative photomicrographs and quantification of infarct size evaluation using Masson Trichrome staining on sections of isotype-treated (White circle) or anti-CD8 depleted (black circle) mice at day 21 after MI. B, Quantification of LV ejection fraction at day 21 by echocardiography, (CTR n=8 and CD8 Depleted n=7). P values were calculated using two-tailed Mann-Whitney test. LV, Left ventricle.

Supplementary Fig. 14

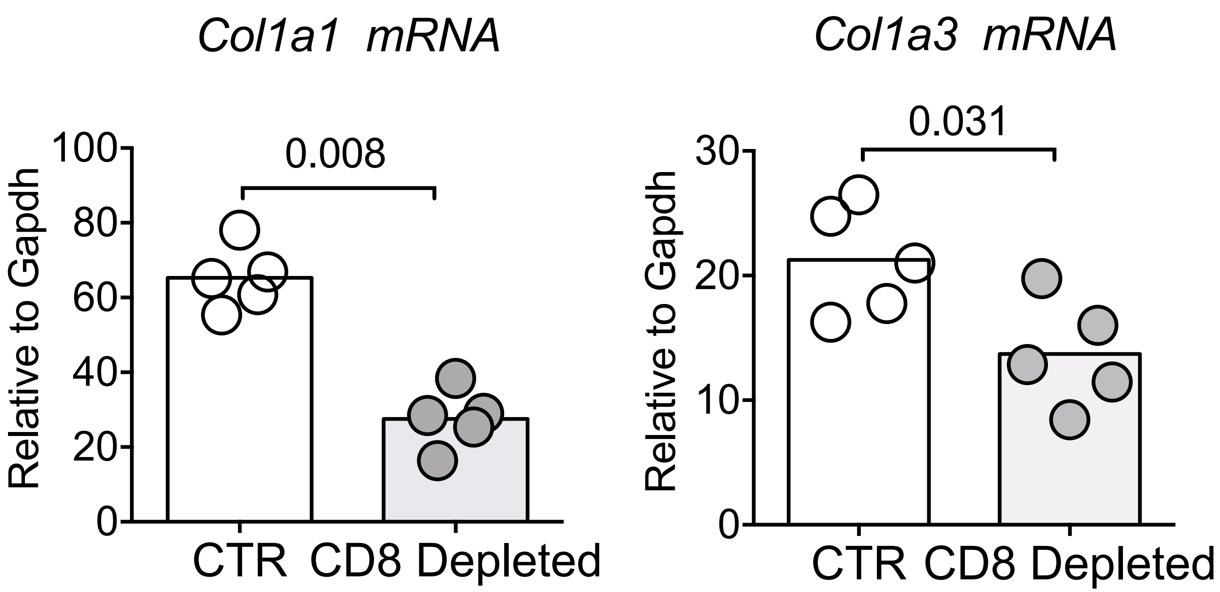

Supplementary Fig.14. CD8⁺ T depletion decreases pro-fibrotic signature in infarcted heart. Quantitative evaluation of *Col1a1 and Col1a3* mRNA levels within the ischemic myocardium, on day 7 after MI in CTR (white) and CD8 Depleted (grey) mice (n=5 per group). P values were calculated using two-tailed Mann-Whitney test.

Supplementary Fig. 15

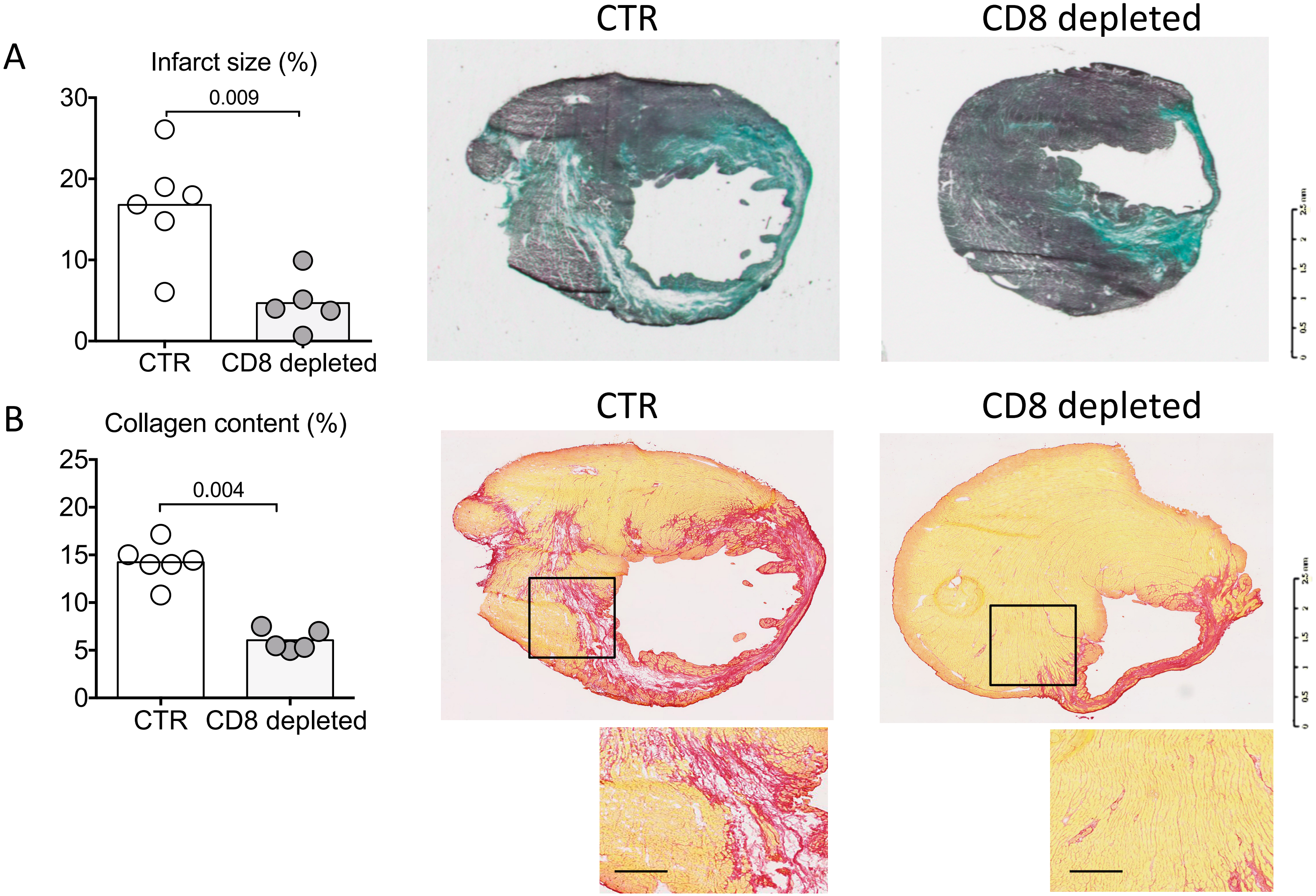

Supplementary Fig.15. The CD8 T cell depleting antibody improves heart function and reduces infarct size at later stage. (a) Representative photomicrographs and quantitative analysis of infarct size evaluation using Masson trichrome staining, in the 2 groups of mice at day 56 after MI. (n=6 CTR white and n= 5 CD8 Depleted Grey). **(b)** Representative photomicrographs and quantitative analysis of myocardial fibrosis evaluated by Sirius Red staining, in the 2 groups of mice at day 56 after MI (n=6 CTR white and n= 5 CD8 Depleted Grey). P values were calculated using two-tailed Mann-Whitney test. bar scale 200 um.

Supplementary Fig. 16

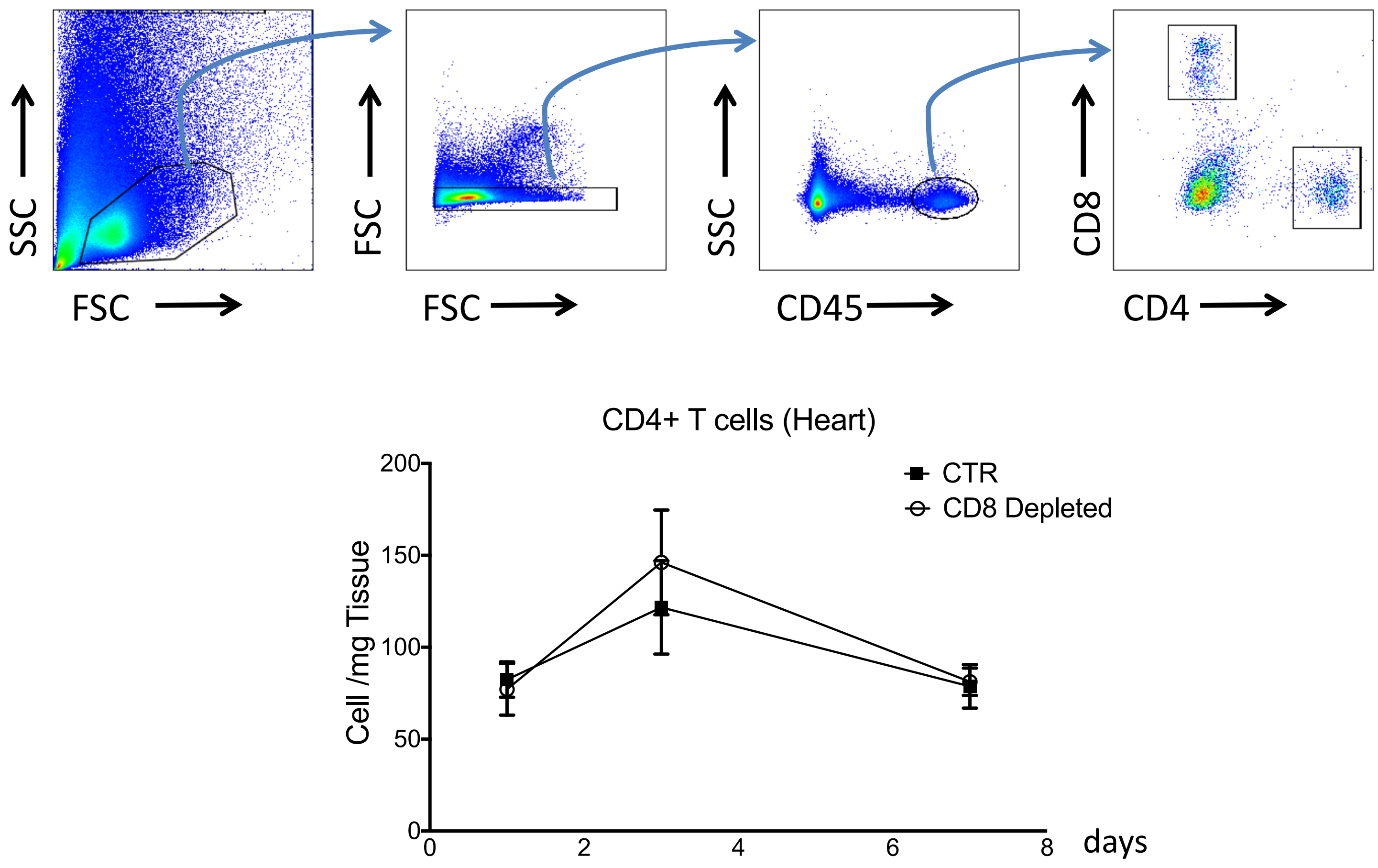

Supplementary Fig.16. The CD8 T cell depleting antibody had no impact on CD4⁺ T cell trafficking in infarcted hearts. Cell suspensions from infarcted hearts of control (Black box) or CD8 depleted (White circle) C57BL/6J mice were stained and analyzed by flow cytometry at different time points after MI. **(a)** CD4+ T lymphocytes were identified as CD45+CD4+ cells. Data are representative of 6 mice per group at each time point. Mean values ± SEM are shown.

Supplementary Fig. 17

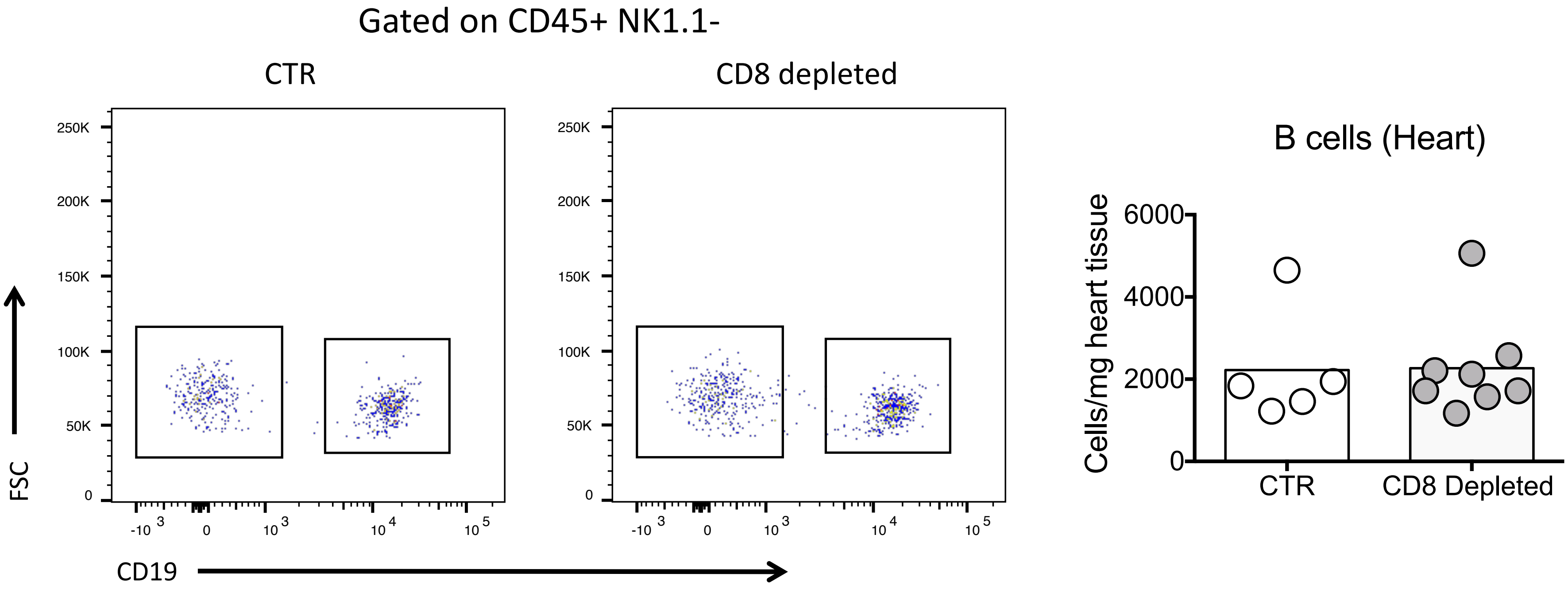

Supplementary Fig.17. The CD8 T cell depleting antibody had no impact on B cell trafficking in infarcted hearts. Cell suspensions from infarcted hearts of control (White) or CD8 depleted (Grey) C57BL/6J mice were stained and analyzed by flow cytometry 5 days after MI. **(a)** B lymphocytes were identified as CD45+NK1.1-CD19+ cells. (N=5-8/group).

Supplementary Fig. 18

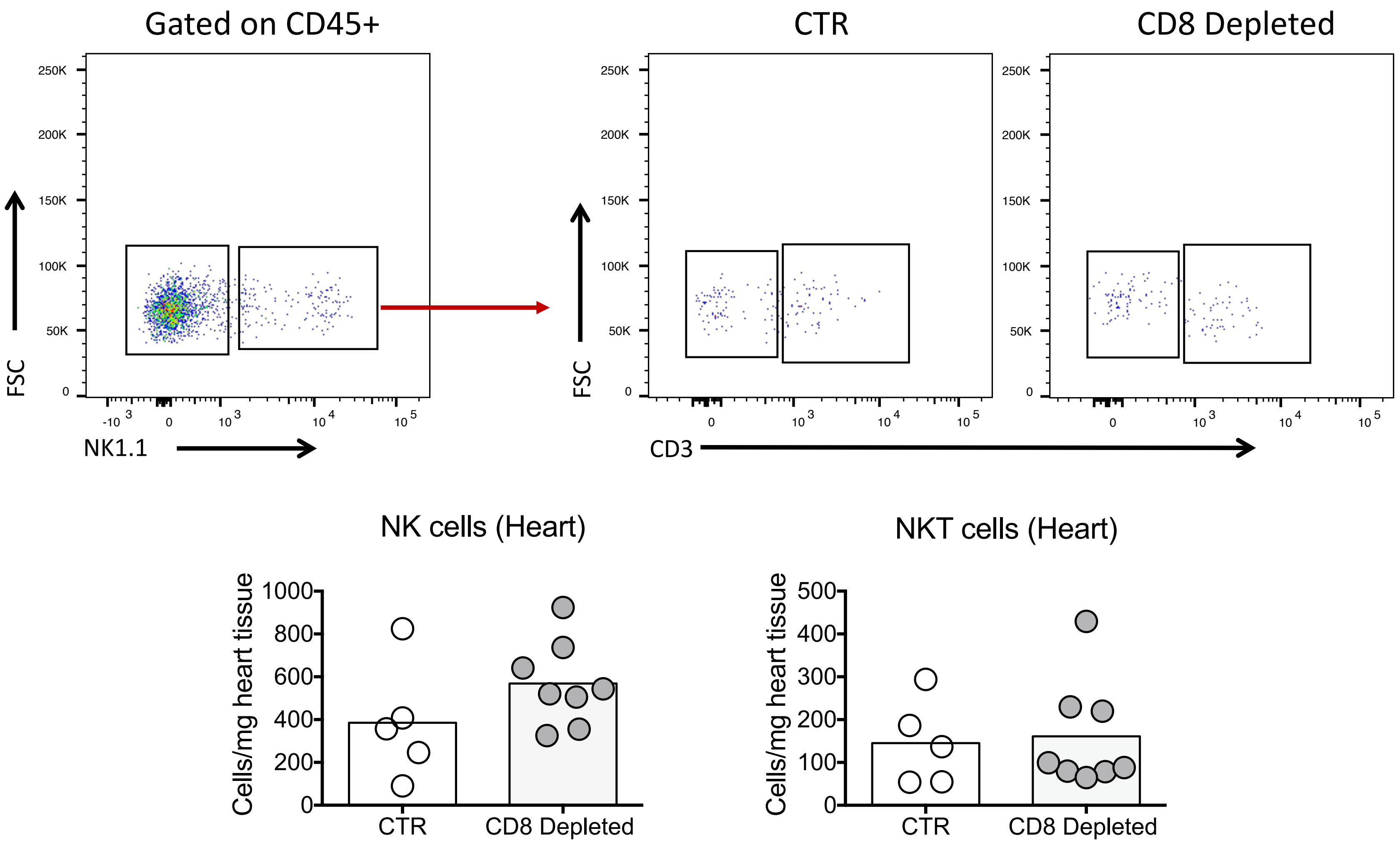

Supplementary Fig.18. The CD8 T cell depleting antibody had no impact on NK and NKT cell trafficking in infarcted hearts. Cell suspensions from infarcted hearts of control (White) or CD8 depleted (Grey) C57BL/6J mice were stained and analyzed by flow cytometry 5 days after MI. **(a)** NKT were identified as CD45+NK1.1+CD3+ cells and NK were identified as CD45+NK1.1+CD3- cells. (N=5-8/group).

Supplementary Fig. 19

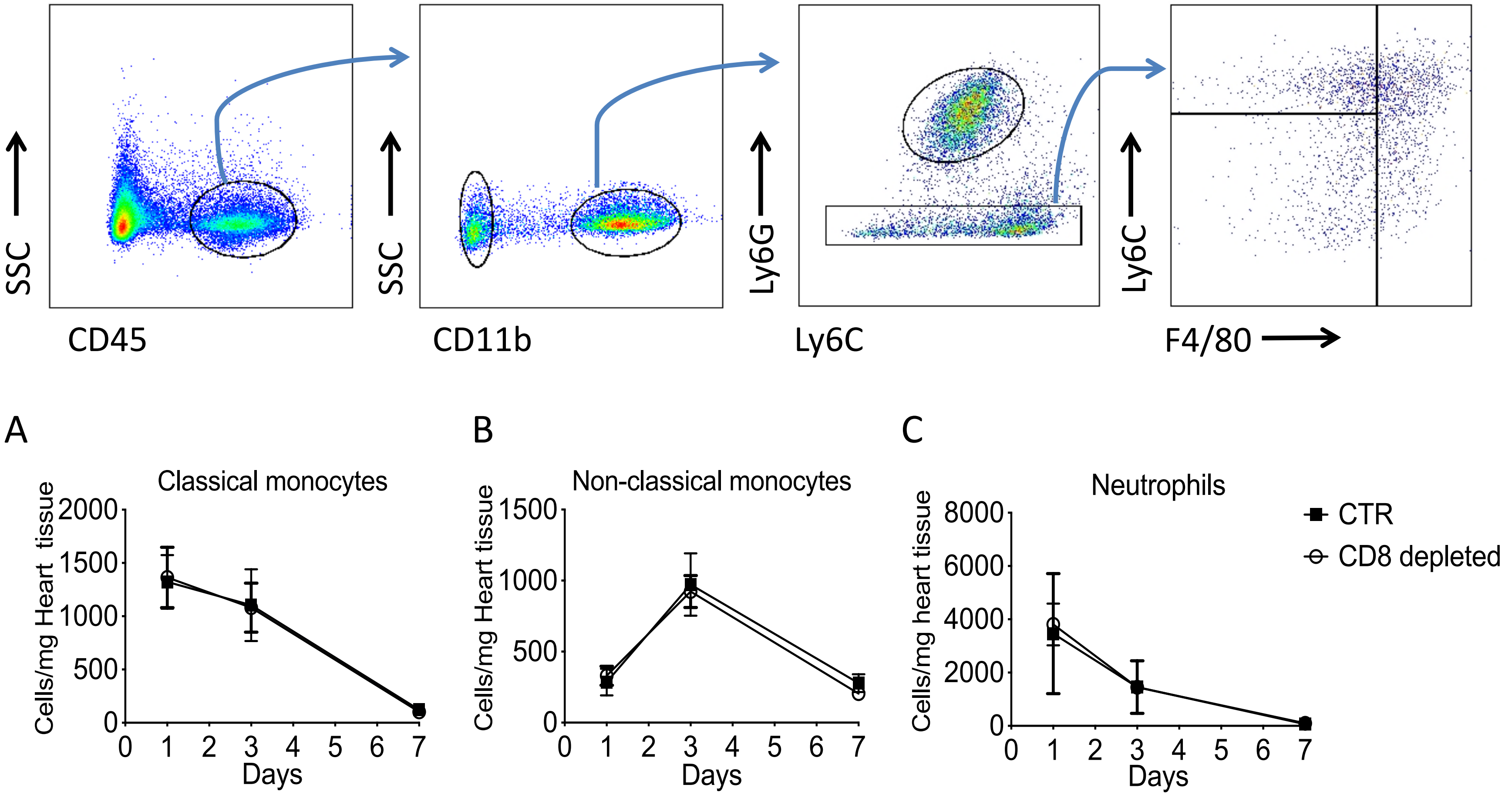

Supplementary Fig.19. The CD8 T cell depleting antibody had no impact on myeloid cell trafficking in infarcted hearts. Cell suspensions from infarcted hearts of control (Black box) or CD8 depleted (White circle) C57BL/6J mice were stained and analyzed by flow cytometry at different time points after MI. **(a)** Classical monocytes were identified as $CD11b^{hi}$ $Ly6G^{—}$ $F4/80^{—}$ $Ly6C^{high}$. **(b)** Non-classical monocytes were identified as $CD11b^{hi}$ $Ly6G^{—}$ $F4/80^{—}$ $Ly6C^{low}$. **(c)** Neutrophils were identified as $CD11b^{+}$ $Ly6G^{+}$ (gated on $CD45^{+}$ cells)Data are representative of 6 mice per group at each time point. Mean values ± SEM are shown.

Supplementary Fig. 20

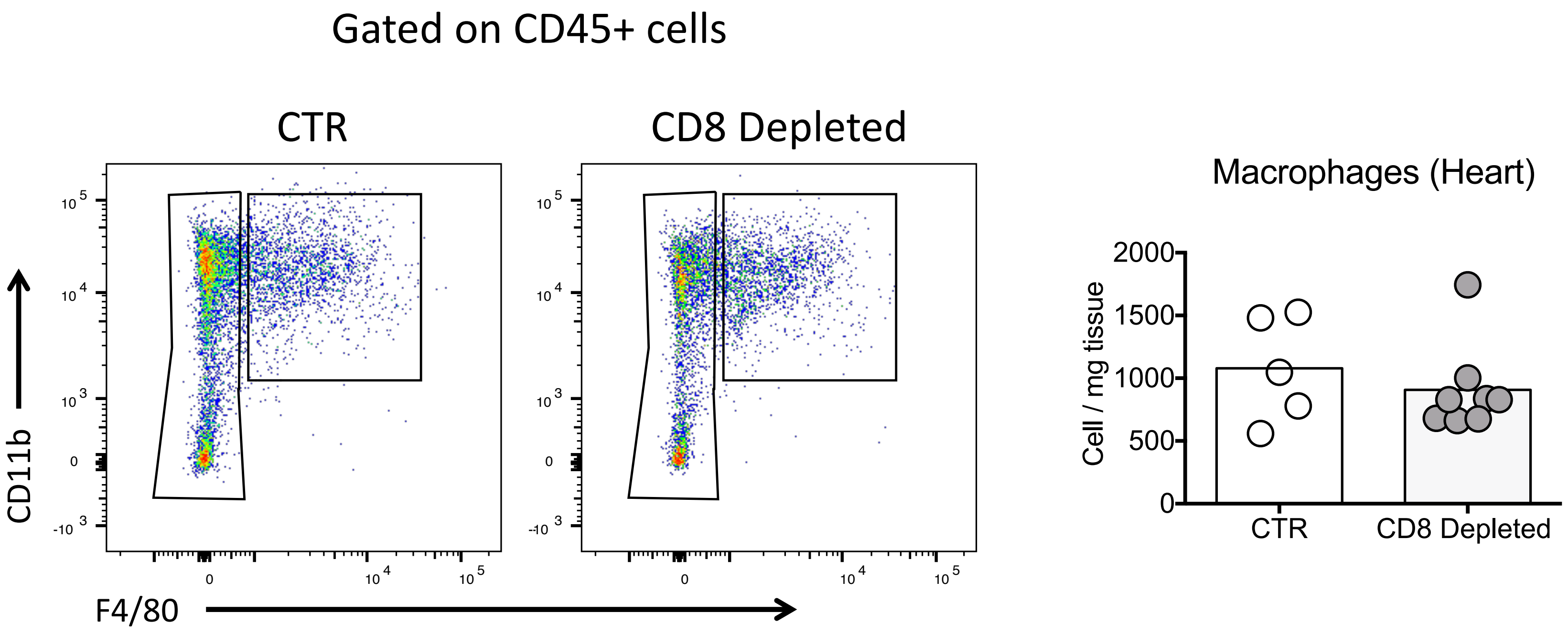

Supplementary Fig.20. The CD8 T cell depleting antibody had no impact on macrophage content in infarcted hearts. Cell suspensions from infarcted hearts of control (White) or CD8 depleted (Grey) C57BL/6J mice were stained and analyzed by flow cytometry 5 days after MI. Macrophages were identified as CD45+CD11b+F4/80+ cells (CTR n=5 and CD8 Depleted n=8).

Supplementary Fig. 21

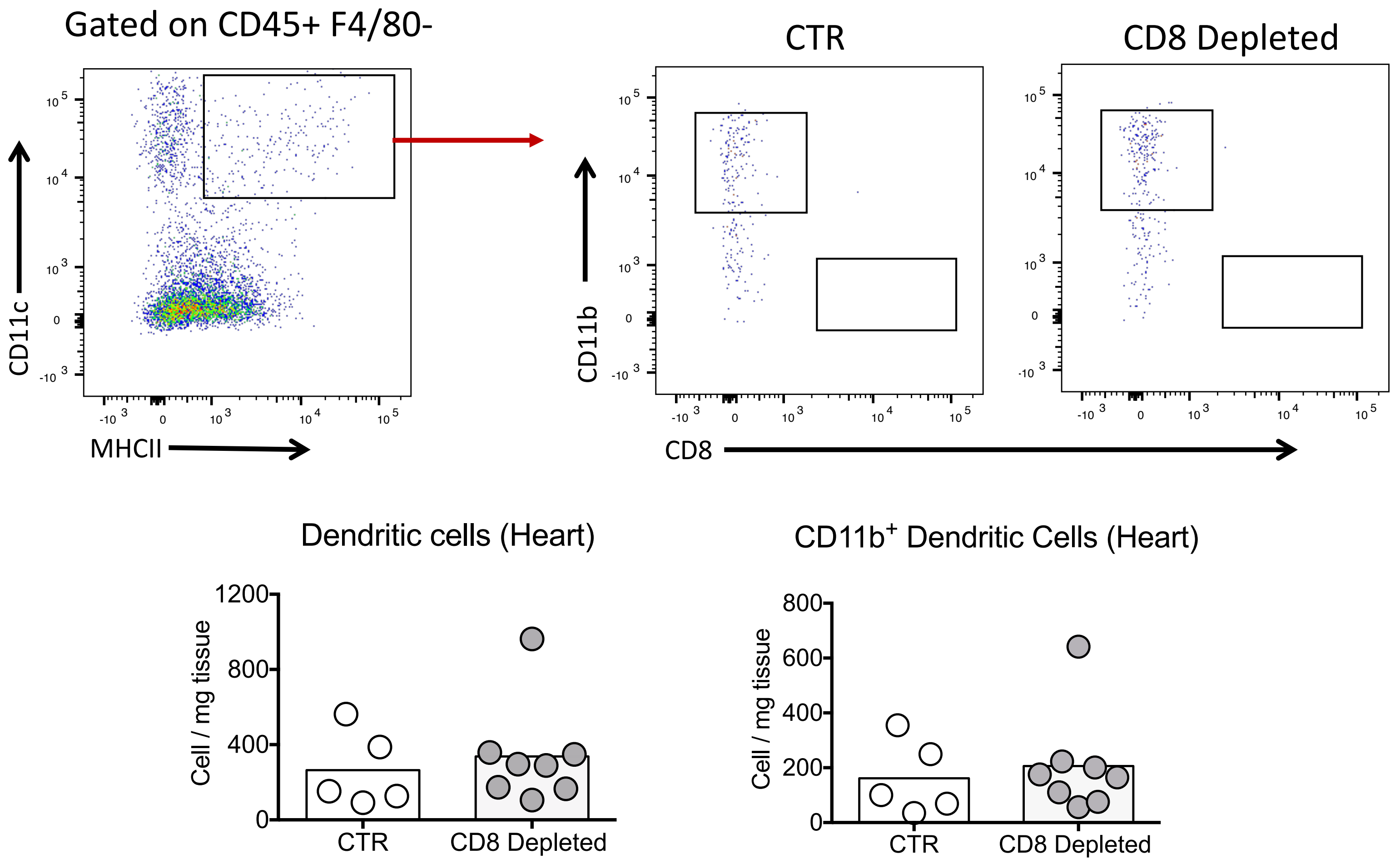

Supplementary Fig.21. The CD8 T cell depleting antibody had no impact on dendritic cell content in infarcted hearts. Cell suspensions from infarcted hearts of control (White) or CD8 depleted (Grey) C57BL/6J mice were stained and analyzed by flow cytometry 5 days after MI. Dendritic cells were identified as CD45+F4/80-CD11c+MHCII+ cells (CTR n=5 and CD8 Depleted n=8).

Supplementary Fig. 22

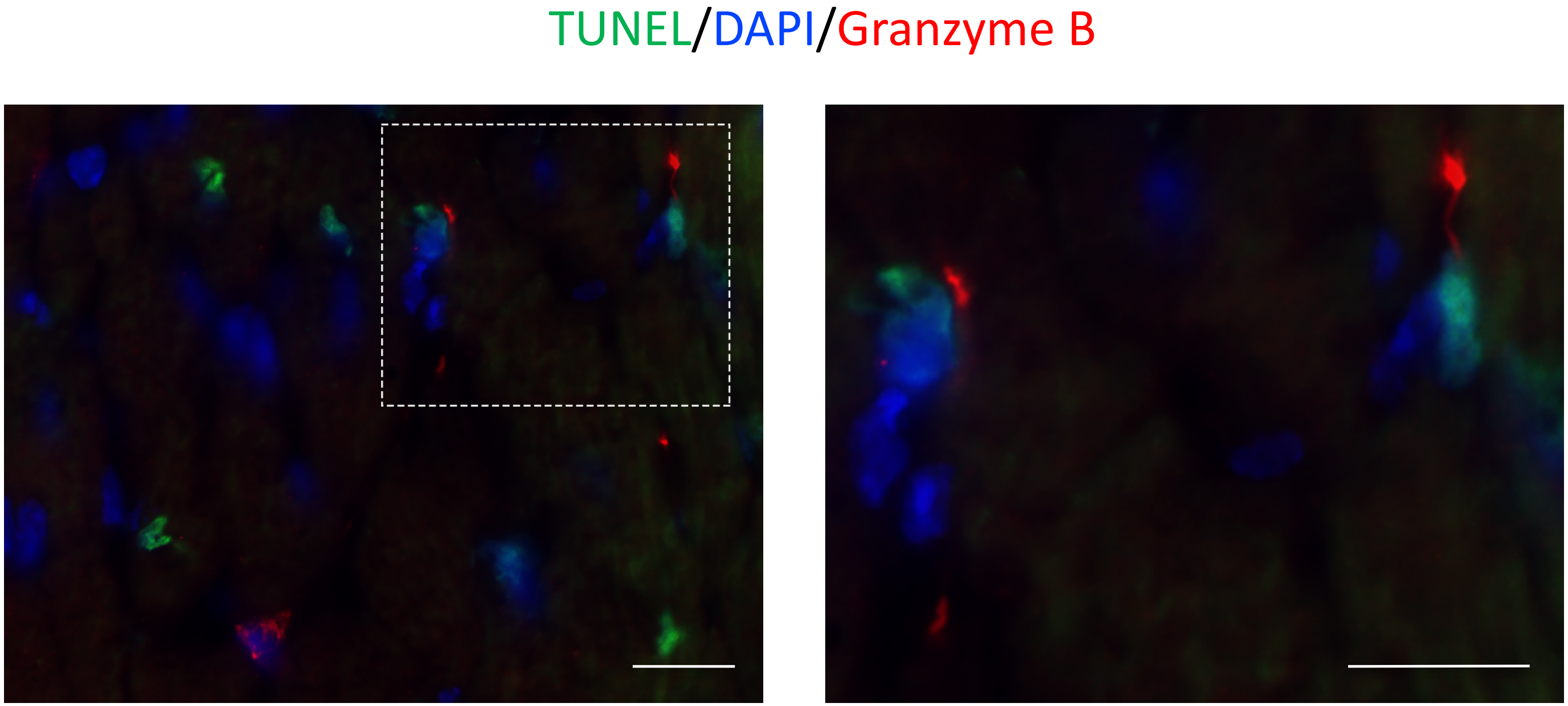

Supplementary Fig.22. Granzyme B colocalizes with apoptotic cells in the infarcted hearts. Granzyme B immunostaining (Red) and TUNEL staining (Green) in the infarcted heart of WT C57BL/6J mice at day 3 after MI. Nuclei were stained using DAPI (Blue). Scale bar 20 μm.

Supplementary Fig. 23

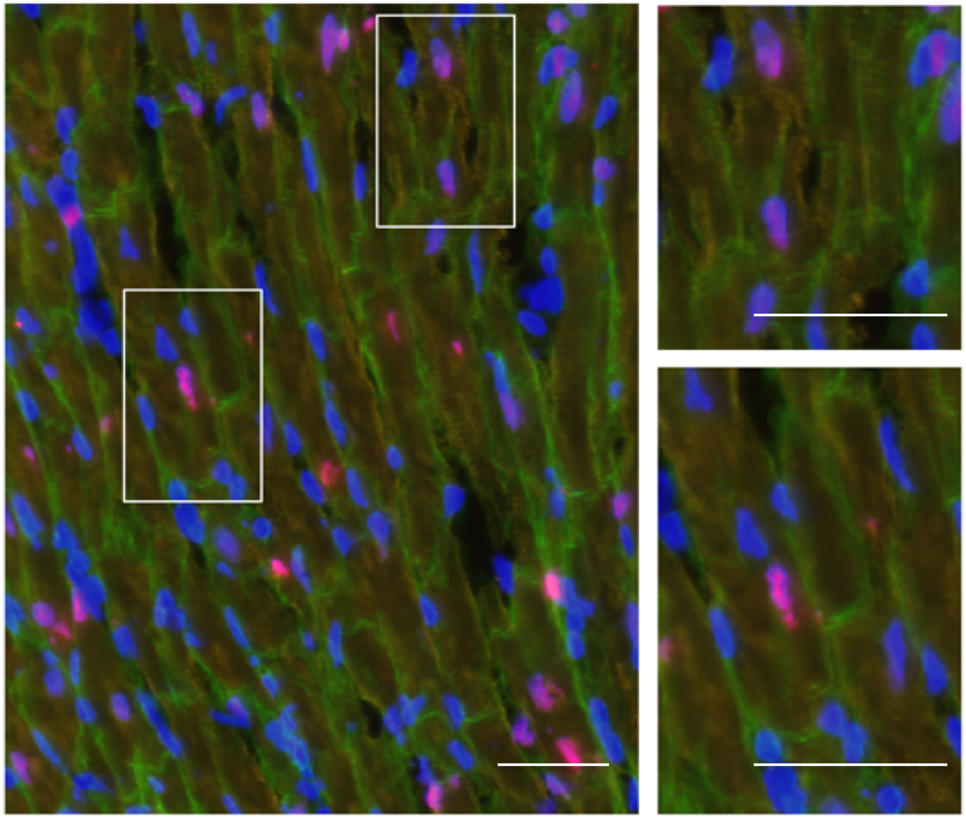

Supplementary Fig.23. Characterization of TUNEL+ cells in ischemic heart tissue. Immunofluorescent staining at day 3 showed that apoptotic cardiomyocytes in ischemic heart tissue. TUNEL (Pink), Troponin T (Brown), Plasma membrane (WGA, Green), and Nuclei (DAPI, Blue). Scale bar 20 μm.

Supplementary Fig. 24

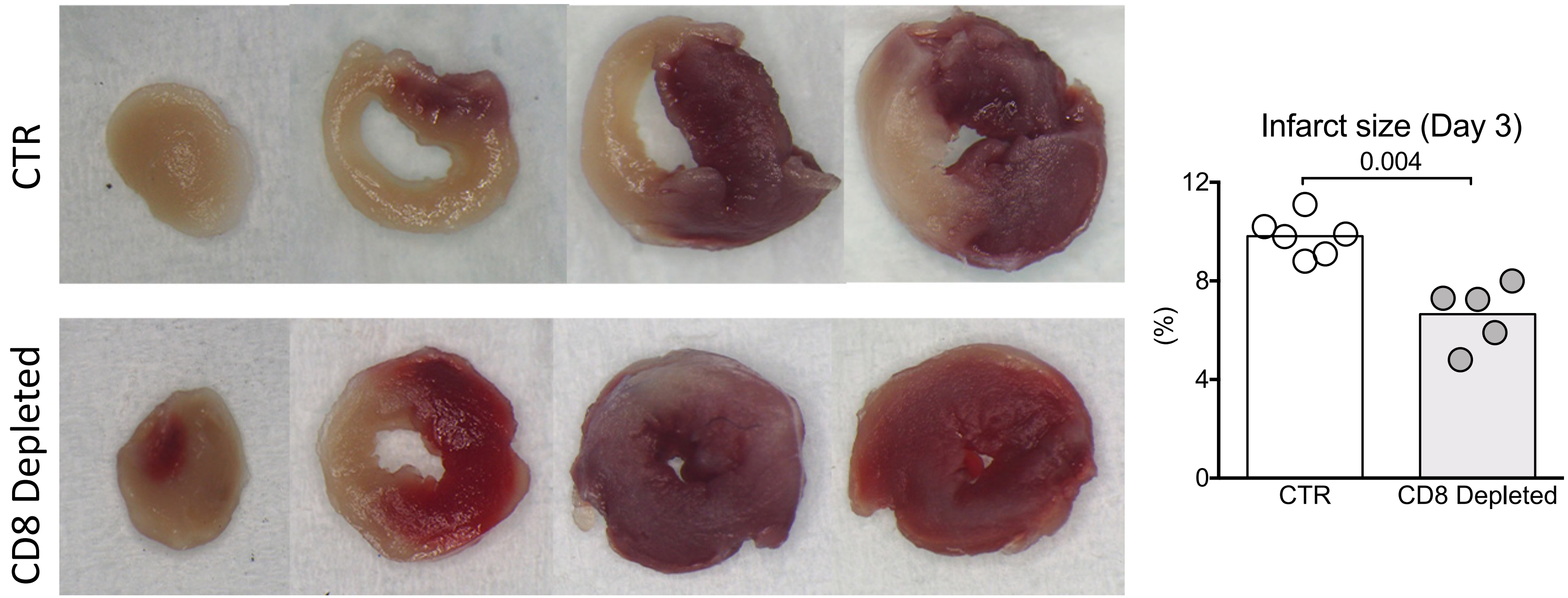

Supplementary Fig.24. The CD8 T cell depleting antibody reduces infarct size at day 3. (a) Representative photomicrographs and quantitative analysis of infarct size evaluation using TriphenylTetrazolium Chloride (TTC) staining, in the 2 groups of mice at day 3 after MI. CTR (White) and CD8 depleted (Grey) (CTR n=6, CD8 Depleted n=5). P values were calculated using two-tailed Mann-Whitney test.

Supplementary Fig. 25

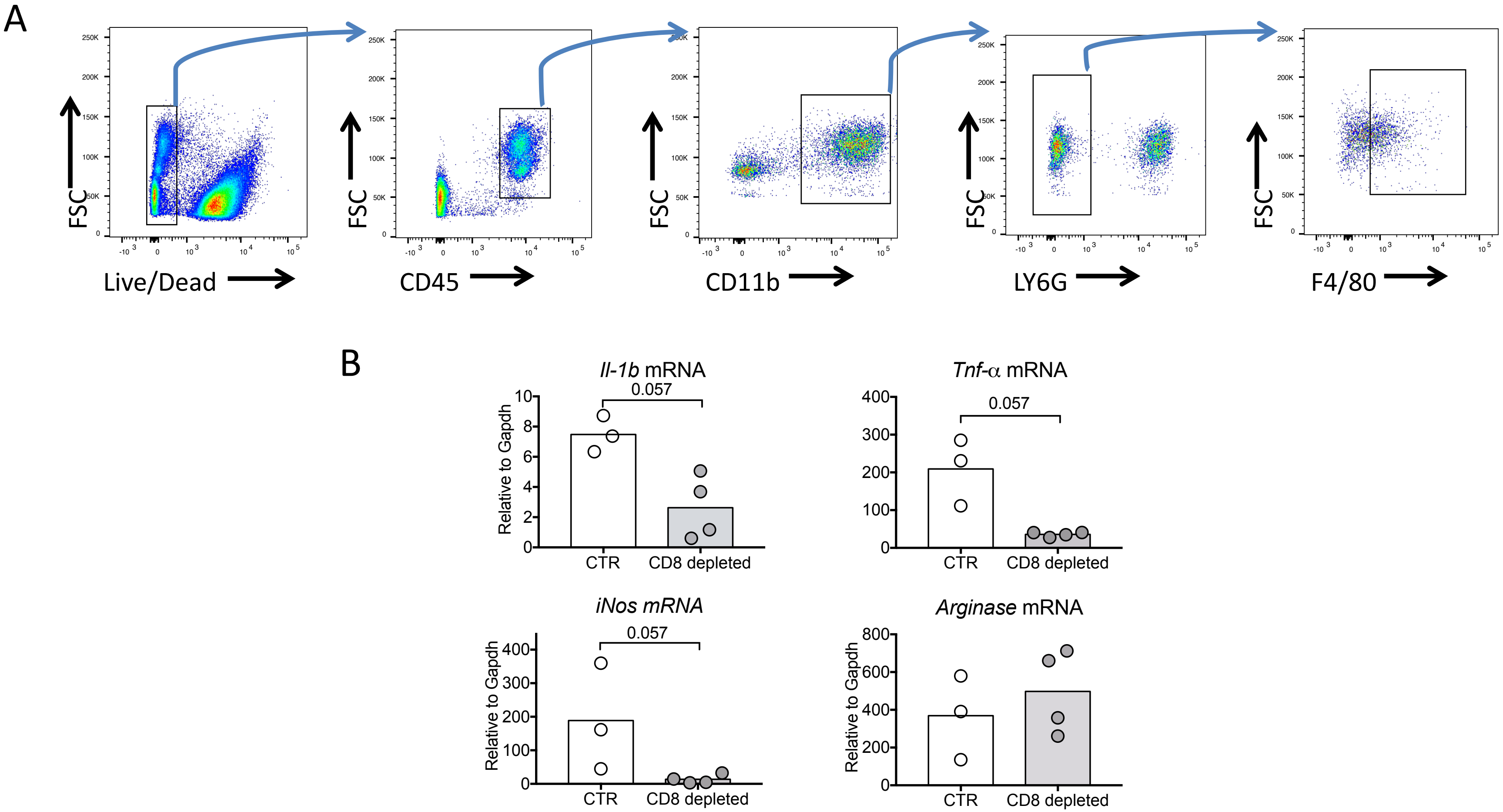

Supplementary Fig.25. The CD8+ T cell depleting antibody promotes a phenotypic switch of tissue macrophages toward a less inflammatory profile. (a) At Day 3 after MI, heart tissue macrophages defined as CD45+CD11B+Ly6G-F4/80+ cells were cell sorted and **(b)** *Il-1β, Tnf-α and iNOS* mRNA levels were measured by qPCR, CTR group (white, n=3) and CD8 depleted (Grey, n=4). P values were calculated using two-tailed Mann-Whitney test.

Supplementary Fig. 26

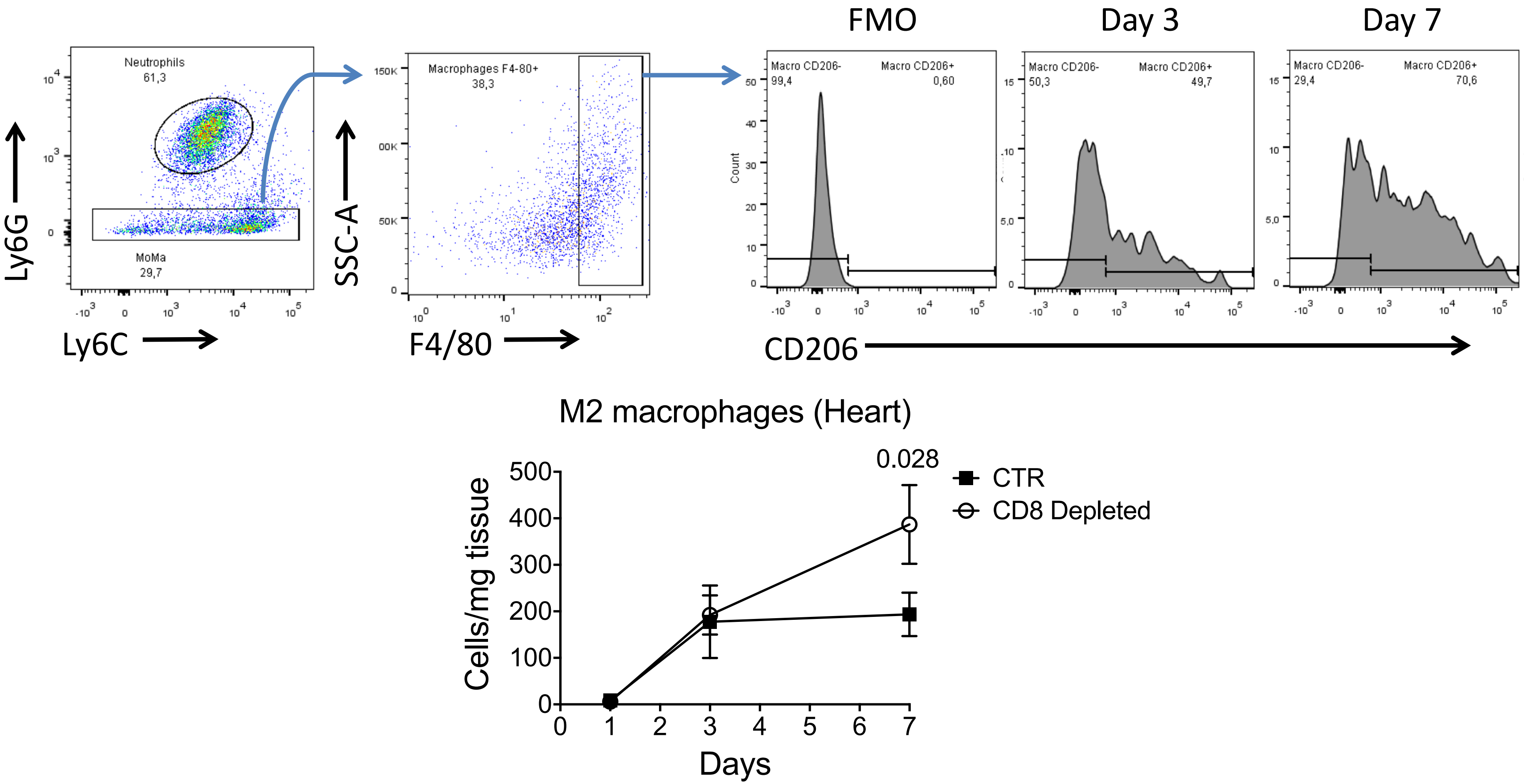

Supplementary Fig.26. The CD8+ T cell depleting antibody promotes a macrophage phenotype associated with alternative activation. Cell suspensions from infarcted hearts of control (Black box) or CD8 depleted (White circle) C57BL/6J mice were stained and analyzed by flow cytometry at different time points after MI. “Alternatively-activated” macrophages were identified as CD45+CD11b+Ly6G— Ly6C+F4/80+CD206+. Data are representative of 6 mice per group at each time point. Mean values ± SEM are shown. P values were calculated using two-tailed Mann-Whitney test at each time point.

Supplementary Fig. 27

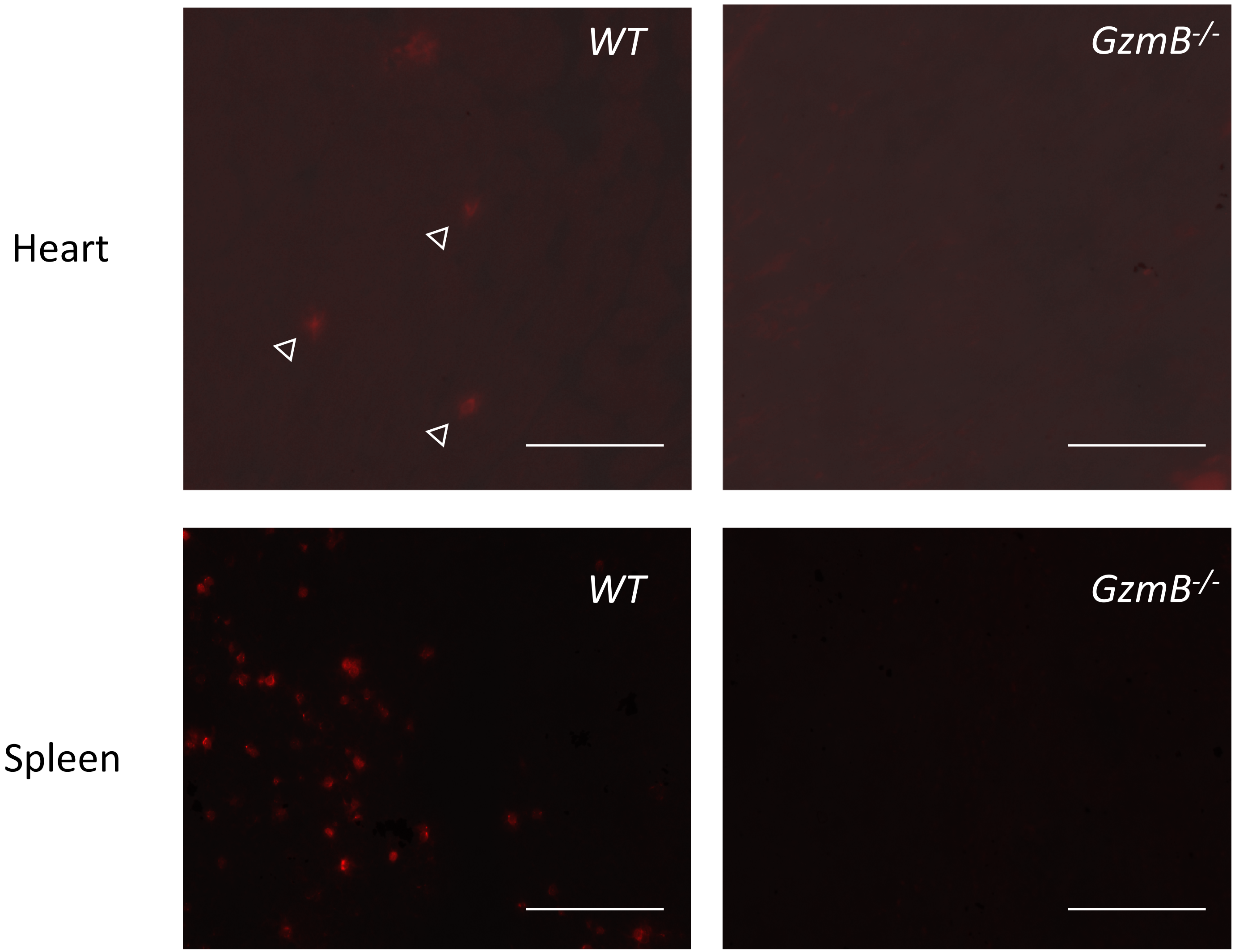

Supplementary Fig.27. Granzyme B is not expressed in the infarcted hearts of *GzmB*$^{-/-}$ mice. Granzyme B immunostaining (Red) in the infarcted heart (Upper panels) and the spleen (Lower panels) of WT C57BL/6J and *GzmB*$^{-/-}$ mice at day 3 after MI. Scale bar 50 μm.

Supplementary Fig. 28

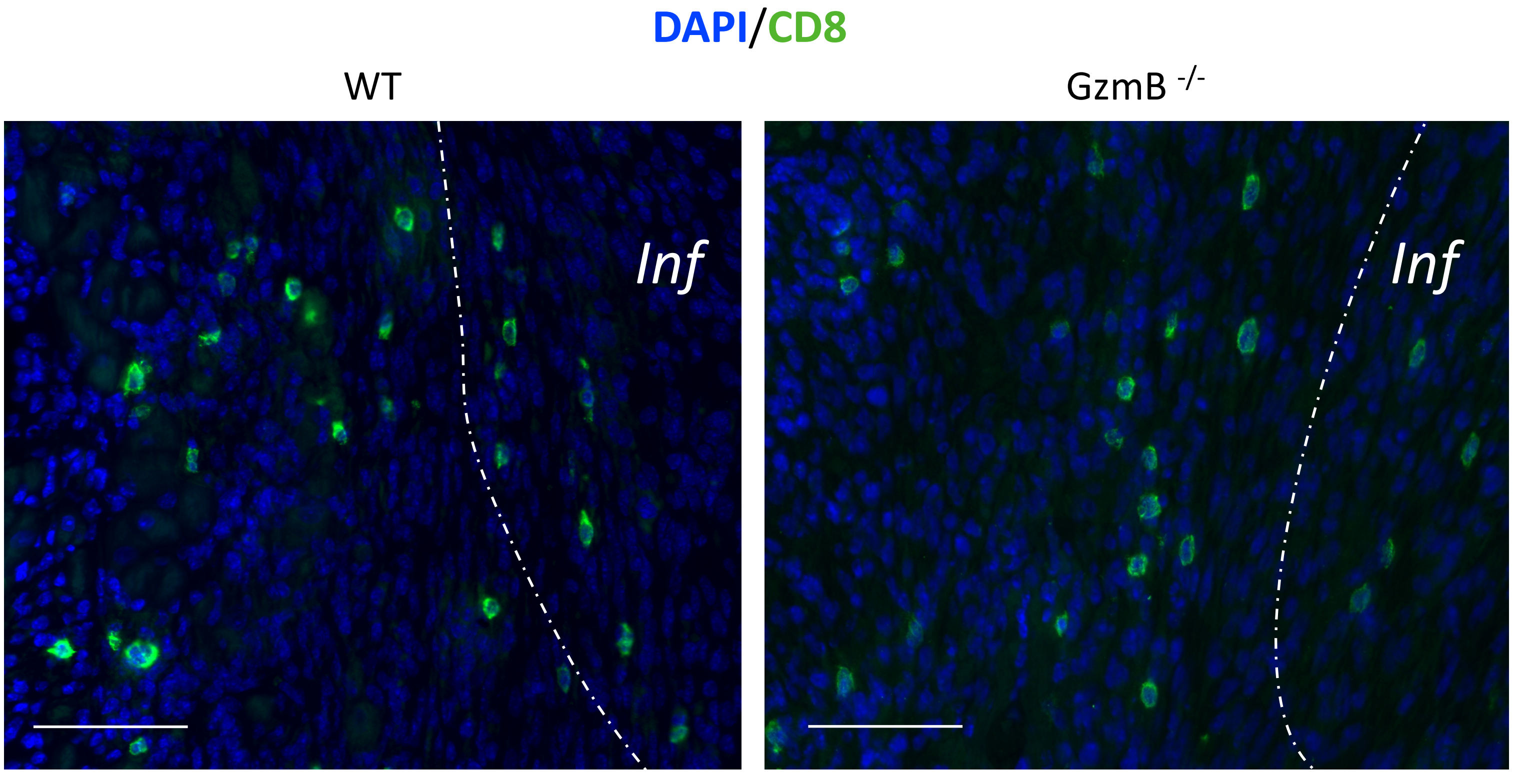

Supplementary Fig.28. *Granzyme B* deficient CD8+ T cells infiltrate ischemic heart. Representative examples of $CD8^+$ T cell infiltration (green) in the heart of C57BL/6J mice or *GzmB*$^{-/-}$ mice at day 3 after MI. *Inf* for infarct area. Scale bar 50 μm.

Supplementary Fig. 29

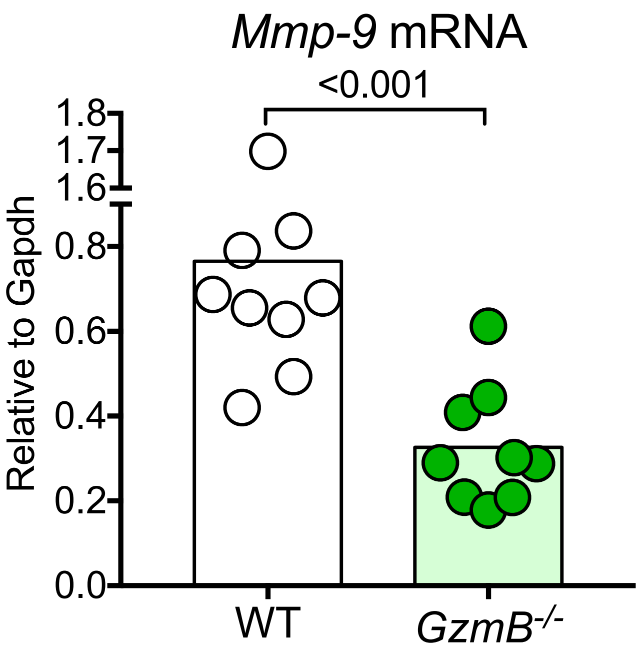

Supplementary Fig.29. Decreased Mmp9 in the infarcted hearts of *GzmB*$^{-/-}$ mice. Mmp9 *mRNA* levels measured by qPCR in infarcted heart at Day 3 after MI, (n=9/group). P values were calculated using two-tailed Mann-Whitney test.

Supplementary Fig. 30

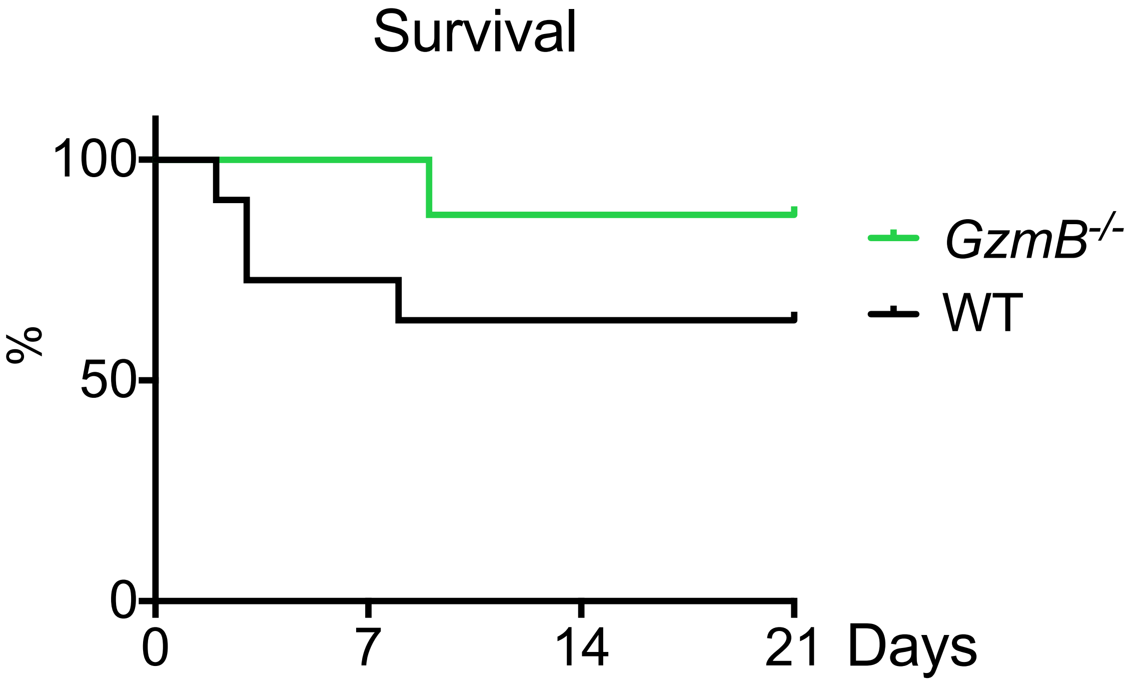

Supplementary Fig.30. Granzyme B deficiency trends to reduce MI-related mortality. Survival curves following acute MI (WT n=11 and *GzmB*$^{-/-}$ n=9).

Supplementary Fig. 31

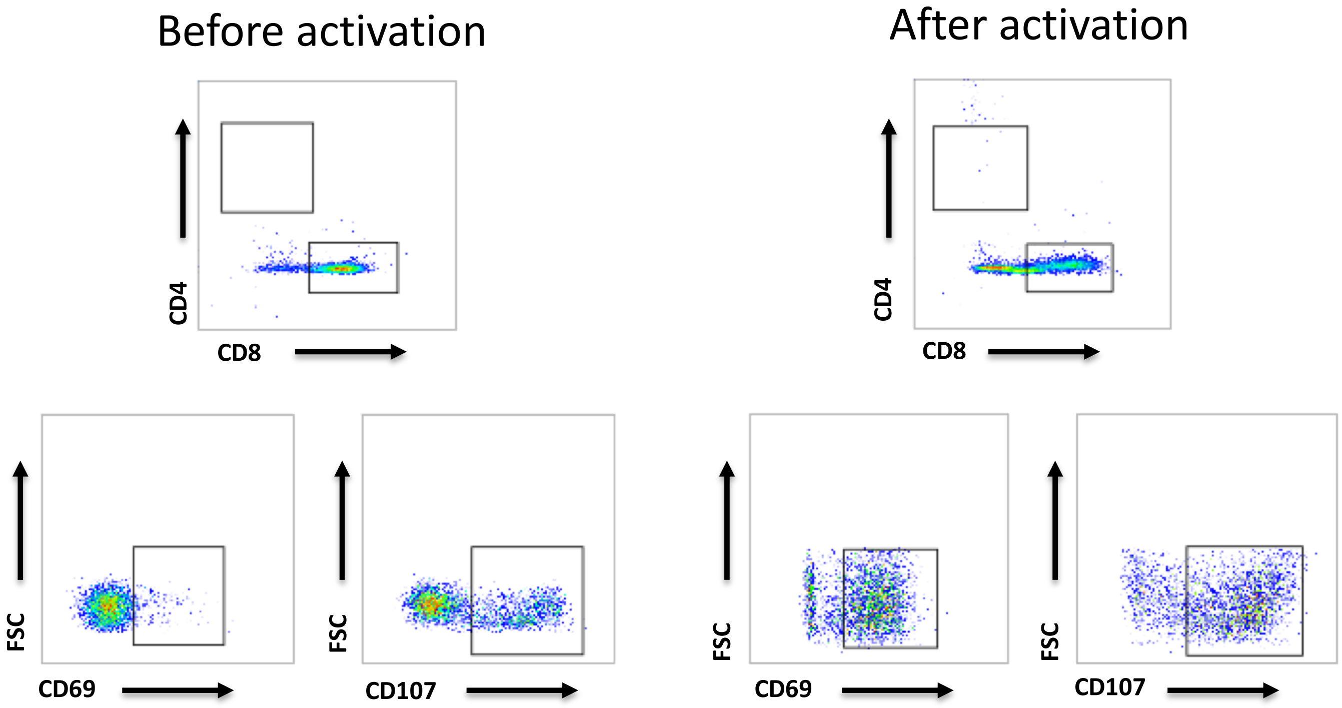

Supplementary Fig.31. Validation of CD8⁺ T cell activation *in vitro*. Flow cytometry characterization of purified splenic CD8⁺ T cells before and after *in vitro* activation.

Supplementary Fig. 32

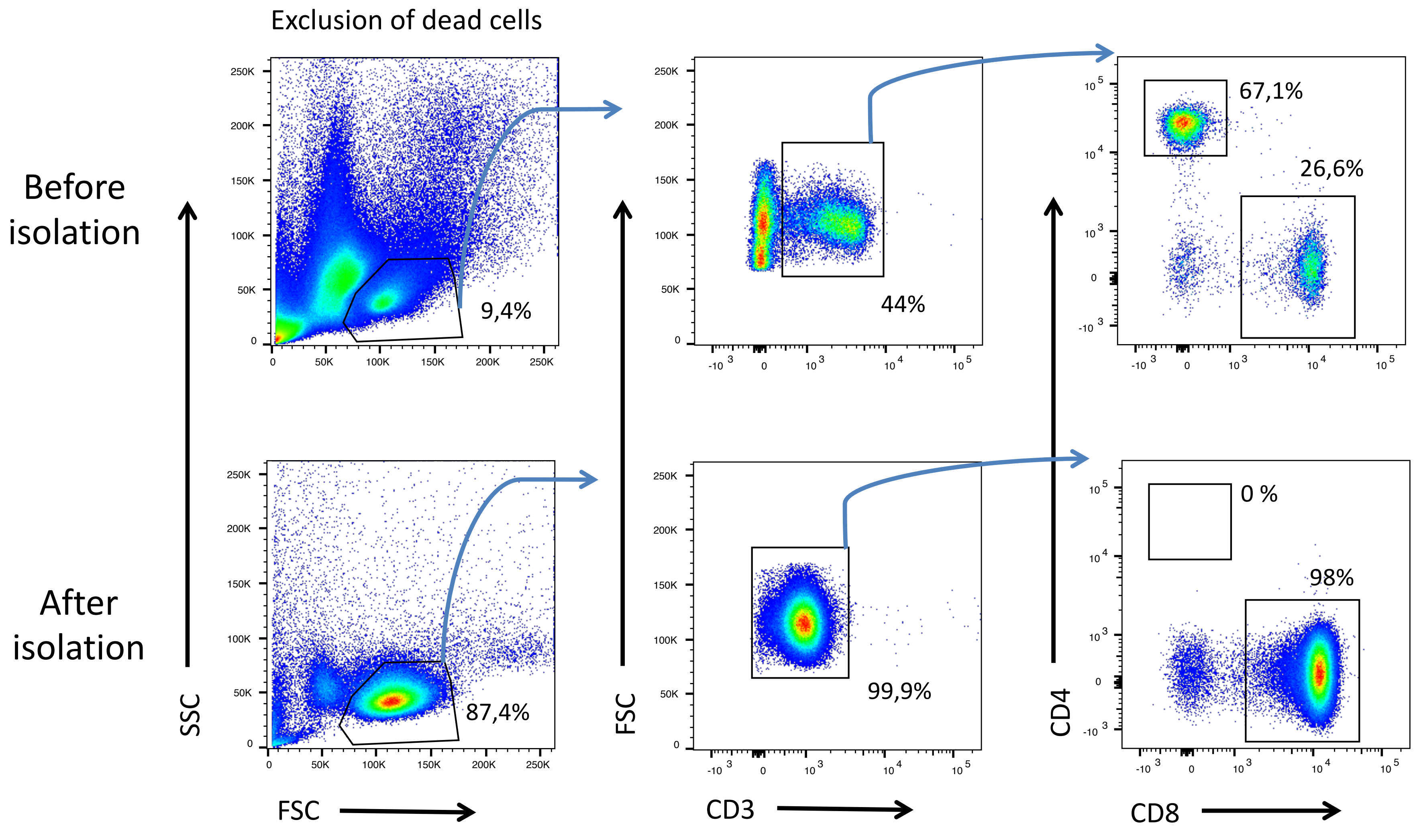

Supplementary Fig.32. CD8⁺ T cell purity after isolation. Representative examples of CD3⁺CD8⁺ T cells before and after purification, using the CD8 cell isolation kit (Miltenyi Biotec) according to manufacturer's instructions.

Supplementary Fig. 33

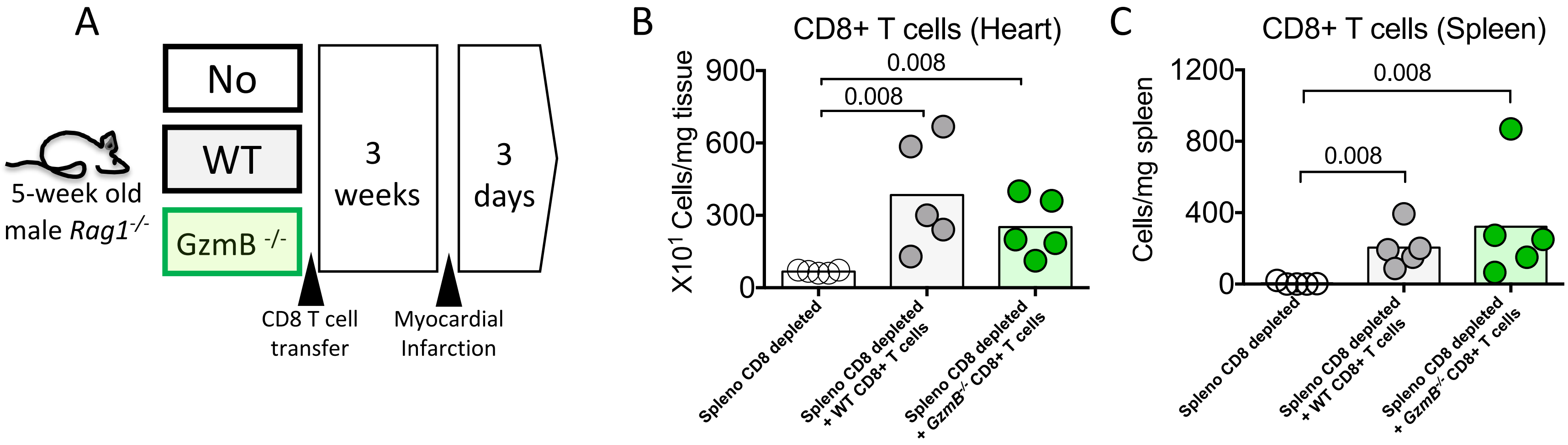

Supplementary Fig.33. Reconstitution of CD8⁺ T cell pool in resupplemented *Rag1⁻/⁻* mice. (a) Experimental design of *Rag1⁻/⁻* mice injected with either CD8-depleted splenocytes (White) or CD8-depleted splenocytes re-supplemented with wild-type (Grey) or *GzmB⁻/⁻* (Green) CD8⁺ T cells, 3 weeks before MI. **(b)** Cell suspensions from infarcted hearts of reconstituted *Rag1⁻/⁻* mice were stained and analyzed by flow cytometry at day 3 after MI. CD8⁺ T cells in the heart were identified as CD45⁺CD3⁺CD8⁺. **(c)** Cell suspensions from spleen of reconstituted *Rag1⁻/⁻* mice were stained and analyzed by flow cytometry at day 3 after MI. CD8+ T cells in the spleen were identified as CD45⁺ CD3⁺ CD8⁺. Data are representative of 5 mice per group at each time point. P values were calculated using two-tailed Kruskal-Wallis test.

Supplementary Fig. 34

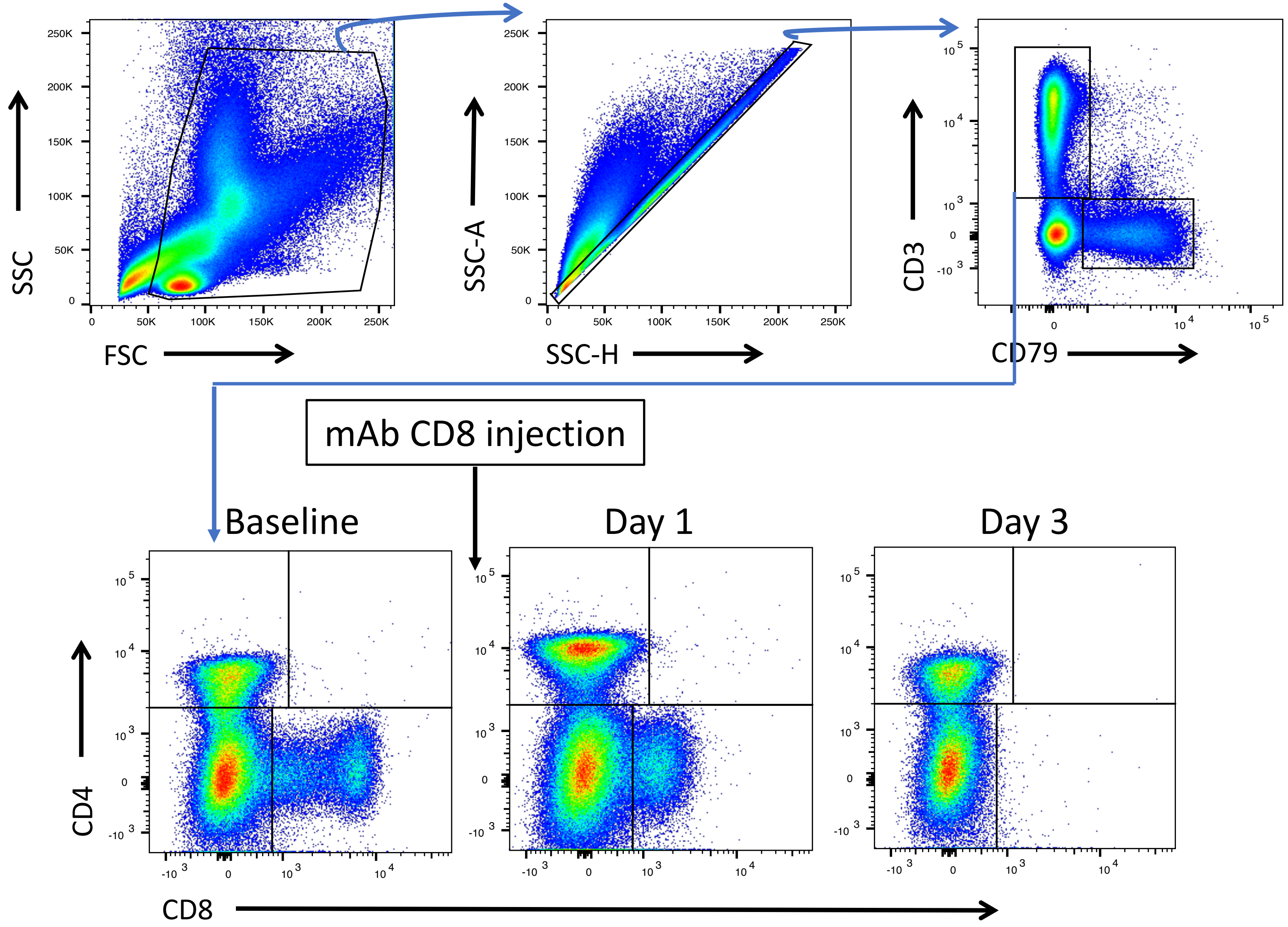

Supplementary Fig.34. Mouse anti-swine mAb CD8 induced delayed CD8 depletion. Blood $CD8^+$ T cells were quantified in the blood at different time points after mAb CD8 injection (15mg/kg, IV) in female pig. CD8+ T cells were defined as CD79-CD3+CD4-CD8+. Cells.

Supplementary Fig. 35

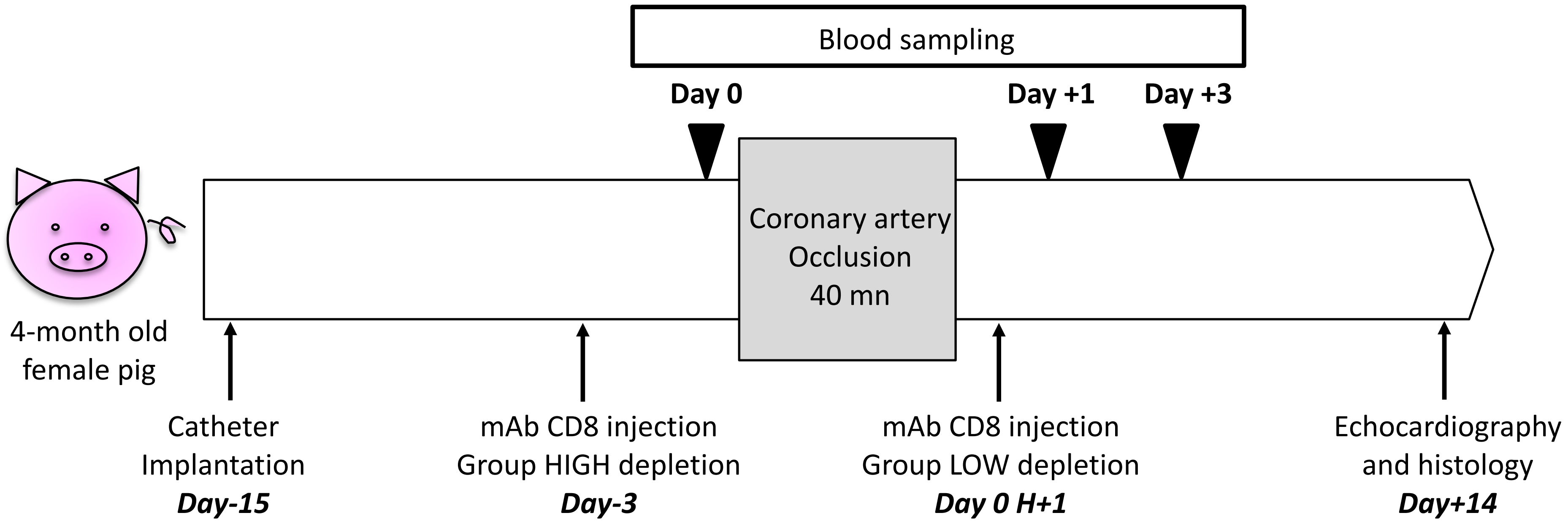

Supplementary Fig.35. Experimental protocol of CD8 depletion in female pigs in the context of myocardial ischemia-reperfusion.

Supplementary Fig. 36

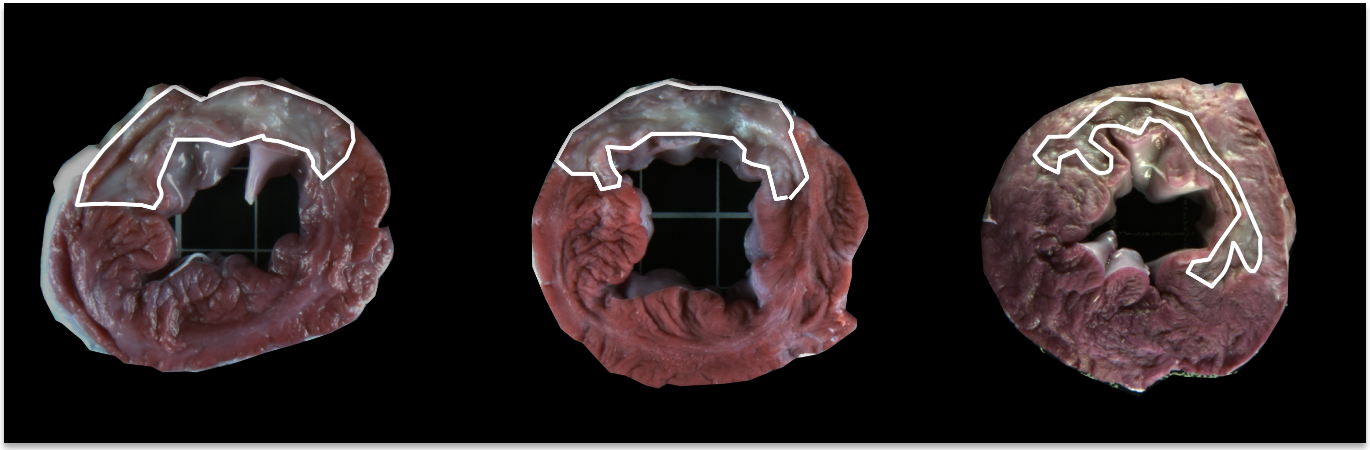

Control

Low CD8 depletion

High CD8 depletion

Supplementary Fig.36. Representative pictures of infarct size at Day 14 in Control, Low and High CD8-depleted groups.

Supplementary Fig. 37

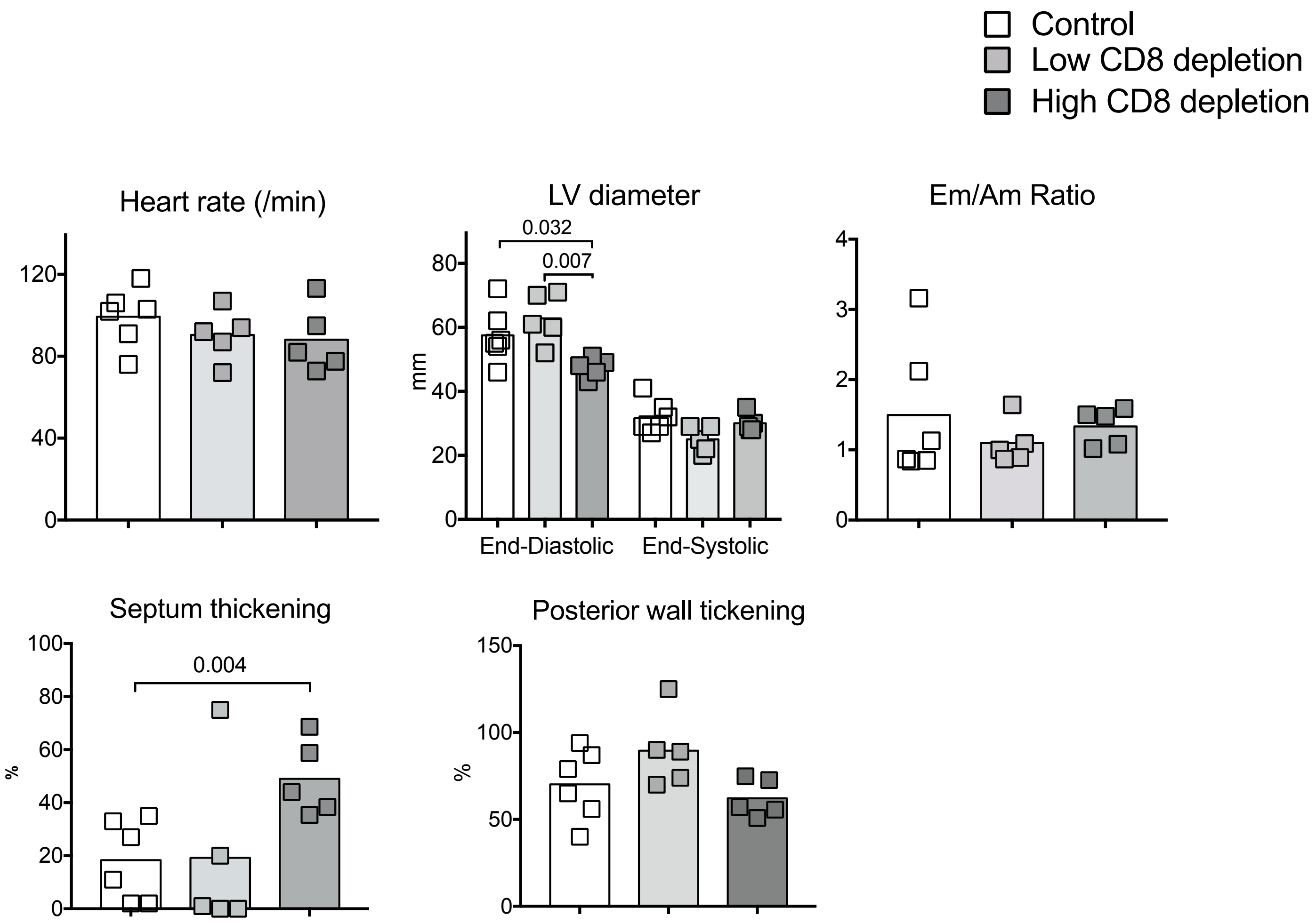

Supplementary Fig.37. Echocardiography parameters at Day 14 in Control (PBS), Low and High CD8-depleted groups (Control n=6, Low depletion n=5 and high depletion n=5). P values were calculated using two-tailed Kruskal-Wallis test. LV, Left ventricle

Supplementary Fig. 38

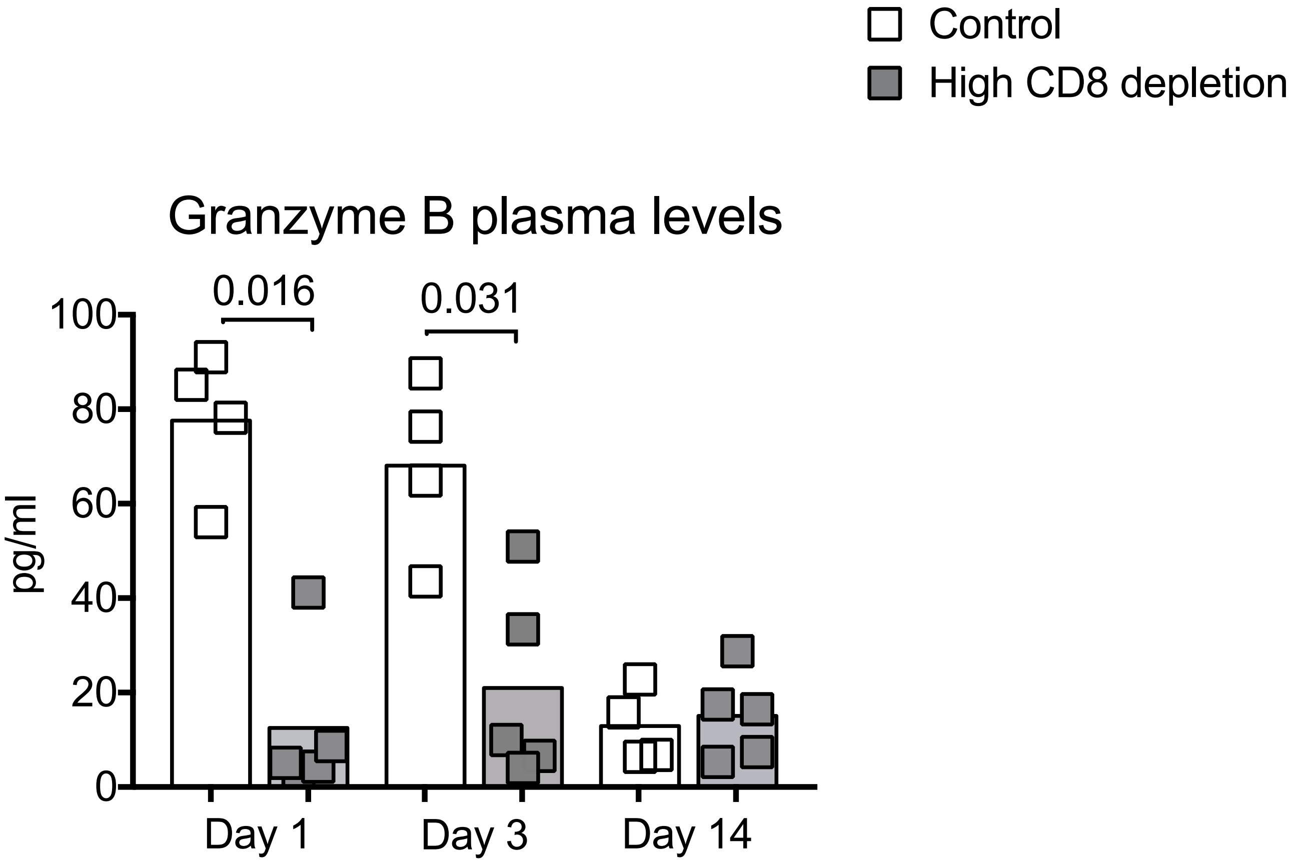

Supplementary Fig.38. Plasma Granzyme B levels in pigs. Plasma Granzyme B levels was measured at Day 1, 3 and 14 after heart ischemia/reperfusion in control (White box, N=4) and CD8 depleted (High depletion, Dark box, N=5) by ELISA. P values were calculated using two-tailed Mann-Whitney test at each timepoint.

**Parameters**	
Myocardial biopsy, n	17
Time between myocardial infarction and LVAD (days)	14 (5;21)
Male Sex	88 %
Age, years	55 (52;61)
Hypertension	41%
Hypercholesterolemia	41%
Diabetus mellitus	23%
Current smokers	59%
Chronic kidney disease	12%
STEMI	88%
No STEMI	12%
PCI	70%
Thrombolyse	0%
Coronary artery bypass surgery	12%
Aspirin	100%
Heparin or Low Molecular Weight Heparin	100%
Cardiogenic shock	100%
Heart transplantation after LVAD	41%

Supplementary Table 1: characteristics of included patients in histopathological study. LVAD, left ventricle assist device; PCI, Percutaneous coronary intervention; STEMI, ST Elevation Myocardial Infarction. Data are expresses as median (1^{st}IQR, 3^{rd}IQR) or percentage.

	< 8.9 pg/mL (N=523)	≥ 8.9 pg/mL (N=523)	p†
Demographic and risk factors			
Male Sex, No (%)	402 (76.9)	388 (74.2)	0.31
Age, yr ‡	63.0 ± 13.4	64.4 ± 14.0	0.11
Hypertension, No (%)	266 (50.9)	276 (52.8)	0.54
Hypercholesterolemia, No (%)	231 (44.2)	229 (43.8)	0.90
Diabetus mellitus, No (%)	111 (21.2)	98 (18.7)	0.31
Family history of CAD, No (%)	152 (29.1)	138 (26.4)	0.33
Current smokers, No (%)	222 (42.4)	191 (36.5)	**0.05**
Prior myocardial infarction, No (%)	69 (13.2)	77 (14.7)	0.48
Prior PCI or CABG, No (%)	73 (14.0)	82 (15.7)	0.43
Prior stroke or TIA, No (%)	18 (3.4)	22 (4.2)	0.52
Prior heart failure, No (%)	11 (2.1)	18 (3.4)	0.19
Chronic kidney disease, No (%)	22 (4.2)	18 (3.4)	0.52
Clinical presentation			
Body mass index, kg/m^2 ‡	26.8 ± 4.6	26.8 ± 4.3	0.94
Systolic blood pressure at admission, mmHg‡	144.8 ± 28.7	146.7 ± 27.0	0.26
Heart rate at admission, beat/min‡	79.2 ± 20.4	77.6 ± 18.4	0.18
STEMI, No (%)	297 (56.8)	269 (51.4)	0.08
STEMI and/or revascularisation, No (%)			0.22
No STEMI	226 (43.2)	254 (48.6)	
STEMI alone	63 (12.0)	55 (10.5)	
STEMI and revascularisation	234 (44.7)	214 (40.9)	
Killip Max >=2, No (%)	86 (16.4)	99 (18.9)	0.29
GRACE score‡	136.8 ± 34.3	137.5 ± 34.6	0.76
Left ventricular ejection fraction, % ‡	51.6 ± 10.5	52.5 ± 11.2	0.17
Baseline biological exams			
CRP, mg/L*	5.0 [3.0 ; 9.8]	5.0 [3.0 ; 10.7]	0.78
In-hospital Management			
PCI, No (%)	420 (80.3)	399 (76.3)	0.12
Thrombolyse, No (%)	45 (8.6)	41 (7.8)	0.65
Coronary artery bypass surgery, No (%)	13 (2.5)	27 (5.2)	**0.02**
Statins, No (%)	475 (90.8)	464 (88.7)	0.26
Beta-blockers, No (%)	431 (82.4)	432 (82.6)	0.94
Calcium channel blockers, No (%)	125 (23.9)	132 (25.2)	0.62
ACE inhibitors or ARB, No (%)	226 (43.2)	244 (46.7)	0.26
Nitrated derivatives, No (%)	251 (48.0)	256 (48.9)	0.76
Aspirin, No (%)	502 (96.0)	513 (98.1)	**0.04**
Clopidogrel, No (%)	403 (77.1)	410 (78.4)	0.60
Heparin, No (%)	231 (44.2)	235 (44.9)	0.80
Low Molecular Weight Heparin, No (%)	293 (56.0)	306 (58.5)	0.42
Diuretics, No (%)	137 (26.2)	157 (30.0)	0.17

Glycoprotein IIb/IIIa inhibitors, No (%)	231 (44.2)	175 (33.5)	**0.0004**
Digitalis glycosides, No (%)	3 (0.6)	4 (0.8)	1
**†p is given by unpaired two-sided Student t or Wilcoxon rank-sum (continuous variables) and exact Pearson X^2 or Fisher exact test (categorical variables)**			
**‡ Mean ± sd, *Median, Q1, Q3**			

Supplementary Table 2: Characteristics of included patients according to baseline plasma Granzyme B level. CAD, Coronary Artery Disease; PCI, Percutaneous coronary intervention; CABG, Coronary By-Pass Graft; TIA, Transient ischemic attack; STEMI, ST Elevation Myocardial Infarction.

Supplementary Table 3

Characteristics	HR	CI 95%	P-value
**Granzyme B median ≥8.9**	2.261	(1.221 - 4.188)	0.0095
**Female gender**	0.879	(0.463 - 1.671)	0.6943
**Age**	1.037	(1.002 - 1.073)	0.0366
**Hypertension**	0.968	(0.492 - 1.905)	0.9253
**BMI >=30**	0.835	(0.395 - 1.766)	0.6370
**Current smokers**	0.953	(0.402 - 2.255)	0.9120
**Hypercholesterolemia**	1.170	(0.665 - 2.056)	0.5859
**Diabetes mellitus**	1.631	(0.873 - 3.048)	0.1249
**STEMI and/or revascularisation**			0.0646
STEMI + revascularisation	0.464	(0.207 - 1.043)	
STEMI without revascularisation	1.490	(0.627 - 3.542)	
No STEMI	1.000		
**Killip Max >=2**	1.495	(0.773 - 2.888)	0.2320
**LV ejection fraction >=40**	1.576	(0.821 - 3.027)	0.1715
**Prior stroke or TIA**	1.535	(0.602 - 3.913)	0.3691
**Prior myocardial infarction**	0.478	(0.217 - 1.053)	0.0671
**Prior heart failure**	7.677	(3.148 - 18.72)	<.0001
**Chronic kidney disease**	1.798	(0.817 - 3.961)	0.1451
**Prior cancer**	2.173	(1.059 - 4.46)	0.0343
**Family history of CAD**	0.785	(0.349 - 1.765)	0.5589
**Statins**	1.571	(0.711 - 3.471)	0.2636
**Beta-blockers**	0.392	(0.218 - 0.704)	0.0017
**Diuretics**	1.068	(0.566 - 2.017)	0.8388
**Clopidogrel**	2.296	(0.912 - 5.779)	0.0776
**Low Molecular Weight Heparin**	0.374	(0.195 - 0.718)	0.0031
**GPIIb/IIIa inhibitors**	0.827	(0.401 - 1.708)	0.6084
**Coronary artery bypass surgery**	0.572	(0.128 - 2.55)	0.4639
**CPK peak**			
CPK > 1701	3.334	(1.082 - 10.279)	0.0360
CPK]595 ; 1701]	1.318	(0.467 - 3.718)	0.6021
CPK]212 ; 595]	0.722	(0.247 - 2.114)	0.5524
CPK Missing	2.935	(1.233 - 6.987)	0.0150
CPK <= 212	1.000		

Supplemental Table 3: Multivariable logistic regression analysis of risk factors for 1-year mortality, using a multivariable Cox proportional-hazards model. HR, Hazard Ratio, BMI, Body Mass Index ; STEMI, ST Elevation Myocardial Infarction ; LV, Left Ventricle ; CAD ; Coronary Artery Disease; CPK peak value was available in 861 patients.

Primary Antibody	Fluorochrome	Clone	Supplier	Reference	Isotype
CD3e	PerCP	145-2C11	BD Biosciences	553067	Armenian Hamster IgG1, K
CD3e	PerCP	145-2C11	eBioscience	45-0031-82	Armenian Hamster IgG1, K
CD3e	BV421	145-2C11	BD Biosciences	562600	Armenian Hamster IgG1, K
CD3e	PE-Cy7	145-2C11	BD Biosciences	552774	Armenian Hamster IgG1, K
CD4	FITC	RM4-5	eBioscience	11-0042-85	Rat IgG2a, k
CD4	PE	RM4-5	eBioscience	12-0043-82	Rat IgG2b, k
CD8a	AF700	53-6.7	BD Biosciences	557959	Rat IgG2a, k
CD8a	APC	53-6.7	BD Biosciences	553035	Rat IgG2a, k
CD45R (B220)	V500	RA3-6B2	BD Biosciences	561226	Rat IgG2a, k
CD19	PE	1D3	eBioscience	12-0193-82	Rat IgG2a, k
CD19	PE-Cy7	1D3	BD Biosciences	552854	Rat IgG2a, k
CD107	APC-Cy7	1D4B	BioLegend	121615	Rat IgG2a, k
CD69	PE	H1.2F3	eBioscience	12-0691-83	Armenian Hamster IgG1, K
CD69	BV421	H1.2F3	BD Biosciences	562920	Armenian Hamster IgG1, K
CD45	FITC	30-F11	BD Biosciences	553079	Rat IgG2b, k
CD45	PerCP	30-F11	BD Biosciences	557235	Rat IgG2b, k
CD45	AF700	30-F11	BD Biosciences	560510	Rat IgG2b, k
CD11b	PE-Cy7	M1/70	eBioscience	25-0112-82	Rat IgG2b, k
CD11c	PerCP-Cy5.5	HL3	BD Biosciences	560584	Armenian Hamster IgG1, K
LY6G	PE	1A8	BD Biosciences	551461	Rat IgG2a, k
LY6C	FITC	AL-21	BD Biosciences	553104	Rat IgM, k
F4/80	APC	A3-1	Bio-Rad	MCA497APCT	Rat IgG2b, k
F4/80	PE	BM8	eBioscience	12-4801-82	Rat IgG2a, k
CD64	BV421	x54-5/7.1	BioLegend	139309	Mouse IgG1, k
CD44	APC	IM7	eBioscience	17-0441-83	Rat IgG2b, k
CD206	AF647	C068C2	BioLegend	141712	Rat IgG2a, k
MHCII	PerCP-eFluor 710	M5/114.15.2	eBioscience	46-5321-82	Rat IgG2b, k
CCR7	PE-Cy7	4B12	eBioscience	17-1971-82	Rat IgG2a, k
NK1.1	PE-Cy7	PK136	eBioscience	25-5941-82	Mouse IgG2a, k
NK1.1	APC	PK136	eBioscience	17-5941-82	Mouse IgG2a, k
CD172A	No Conjugated	-	Monoclonal Antibody Center	PG2031	IgG2b
MSA3 (MHCII)	No Conjugated	-	Monoclonal Antibody Center	PG2006	IgG2a
PG68A	No Conjugated	-	Monoclonal Antibody Center	PG2045	IgG1
SWC8	No Conjugated	MIL3	Abcam	Ab34020	IgGM
CD14	FITC	TUK4	GENE TEX	GTX43753	Mouse IgG2a
CD163	PE	2A10/11	Abcam	Ab194889	Mouse IgG1
PG164A (CD8b)	No Conjugated	-	Monoclonal Antibody Center	PG2020	IgG2a
CD4	PE-Cy7	74-124	Abcam	Ab25408	Mouse IgG2a
CD3e	PerCP-Cy5.5	BB23-8E6-8C8	BD Biosciences	561478	Mouse IgG2a, k
CD79a	PE	HM57	BD Biosciences	563777	Mouse IgG1

Supplementary Table 4. General characteristics of antibodies used for flow cytometry characterization of immune cells.

Secondary Antibody	Fluorochrome	Clone	Supplier	Reference
**Goat Anti-Mouse IgG2b**	APC-Cy7	Polyclonal	Abcam	ab130791
**Rat Anti-Mouse IgG2a**	PE-Cy7	RMG2a-62	BioLegend	407114
**Rat Anti-Mouse IgG1**	PerCP-Cy5.5	RMG1-1	BioLegend	406612
**Rat Anti-mouse IgM**	APC	Nov-41	BD Biosciences	550676

Supplementary Table 5. General characteristics of secondary antibodies used for flow cytometry characterization of immune cells.

Genes	Sequences
Gapdh	Forward 5′-CGTCCCGTAGACAAAATGGTGAA-3′,<br>Reverse 5′-GCCGTGAGTGGAGTCATACTGGAA-CA-3′;
Granzyme B	Forward 5′-GTGCGGGGGACCCAAAGACCAAAC-3′,<br>Reverse: 5′-GCACGTGGAGGTGAACCATCCTTATAT-3′
Il1β	Forward 5’-GAAGAGCCCATCCTCTGTGA-3’,<br>Reverse 5’-GGGTGTGCCGTCTTTCATTA-3’
Il6	Forward 5′-TGACAACCACGGCCTTCCCTA-3′,<br>Reverse: 5′-TCAGAATTGCCATTGCACAACTCTT-3’
Il10	Forward 5′-ACTTCCCAGTCGGCCAGAGCCACAT-3′,<br>Reverse: 5′-GATGACAGCGCCTCAGCCGCATCCT-3′
Tnf-α	Forward 5’-GATGGGGGGCTTCCAGAACT-3’,<br>Reverse 5’-GATGGGGGGCTTCCAGAACT-3’
Mmp9	Forward 5’-GCGTCATTCGCGTGGATAAGGAGT-3’,<br>Reverse 5’-GTAGCCCACGTCGTCCACCTGGTT-3’

Supplementary Table 6. The primer sequences used for quantitative real-time PCR.
