# Supplementary material for: Cytotoxic CD8+ T cells promote granzyme B-dependent adverse post-ischemic cardiac remodeling
Source: Nat Commun. 2021 Mar 5;12:1483. doi: 10.1038/s41467-021-21737-9 (PMC7935973; doi:10.1038/s41467-021-21737-9)
Supplement: Supplementary file 3 — Reporting Summary [file 41467_2021_21737_MOESM3_ESM.pdf]

nature research

Corresponding author(s): Ait-Oufella

Last updated by author(s): Jan 18, 2021

Reporting Summary

Nature Research wishes to improve the reproducibility of the work that we publish. This form provides structure for consistency and transparency in reporting. For further information on Nature Research policies, see our Editorial Policies and the Editorial Policy Checklist.

Statistics

For all statistical analyses, confirm that the following items are present in the figure legend, table legend, main text, or Methods section.

n/a	Confirmed	
☐	☒	The exact sample size (*n*) for each experimental group/condition, given as a discrete number and unit of measurement
☐	☒	A statement on whether measurements were taken from distinct samples or whether the same sample was measured repeatedly
☐	☒	The statistical test(s) used AND whether they are one- or two-sided<br>*Only common tests should be described solely by name; describe more complex techniques in the Methods section.*
☐	☒	A description of all covariates tested
☐	☒	A description of any assumptions or corrections, such as tests of normality and adjustment for multiple comparisons
☐	☒	A full description of the statistical parameters including central tendency (e.g. means) or other basic estimates (e.g. regression coefficient) AND variation (e.g. standard deviation) or associated estimates of uncertainty (e.g. confidence intervals)
☐	☒	For null hypothesis testing, the test statistic (e.g. *F*, *t*, *r*) with confidence intervals, effect sizes, degrees of freedom and *P* value noted<br>*Give P values as exact values whenever suitable.*
☒	☐	For Bayesian analysis, information on the choice of priors and Markov chain Monte Carlo settings
☒	☐	For hierarchical and complex designs, identification of the appropriate level for tests and full reporting of outcomes
☐	☒	Estimates of effect sizes (e.g. Cohen's *d*, Pearson's *r*), indicating how they were calculated

Our web collection on statistics for biologists contains articles on many of the points above.

Software and code

Policy information about availability of computer code

Data collection	material and methods
Data analysis	Histology: ImageJ<br>Flow cytometry : FlowJoSoftware (TreeStar)<br>Statistics: SAS software version 9.4

For manuscripts utilizing custom algorithms or software that are central to the research but not yet described in published literature, software must be made available to editors and reviewers. We strongly encourage code deposition in a community repository (e.g. GitHub). See the Nature Research guidelines for submitting code & software for further information.

Data

Policy information about availability of data

All manuscripts must include a data availability statement. This statement should provide the following information, where applicable:

- Accession codes, unique identifiers, or web links for publicly available datasets
- A list of figures that have associated raw data
- A description of any restrictions on data availability

The data that support the findings of this study are available from the corresponding author on reasonable request.

Field-specific reporting

Please select the one below that is the best fit for your research. If you are not sure, read the appropriate sections before making your selection.

- [x] Life sciences
- [ ] Behavioural & social sciences
- [ ] Ecological, evolutionary & environmental sciences

For a reference copy of the document with all sections, see nature.com/documents/nr-reporting-summary-flat.pdf

Life sciences study design

All studies must disclose on these points even when the disclosure is negative.

Sample size	Sample size calculation was not performed and number of mice in each experiments was based on our expertise and previous studies we performed on experimental myocardial infarction
Data exclusions	No
Replication	Yes for qualitative analysis (immunostainings)
Randomization	No because of organization issue in our facilities but analysis were done blindly
Blinding	all analysis were done blindly

Reporting for specific materials, systems and methods

We require information from authors about some types of materials, experimental systems and methods used in many studies. Here, indicate whether each material, system or method listed is relevant to your study. If you are not sure if a list item applies to your research, read the appropriate section before selecting a response.

Materials & experimental systems

n/a	Involved in the study	
[ ]	[x]	Antibodies
[x]	[ ]	Eukaryotic cell lines
[x]	[ ]	Palaeontology and archaeology
[ ]	[x]	Animals and other organisms
[ ]	[x]	Human research participants
[ ]	[x]	Clinical data
[x]	[ ]	Dual use research of concern

Methods

n/a	Involved in the study	
[x]	[ ]	ChIP-seq
[ ]	[x]	Flow cytometry
[x]	[ ]	MRI-based neuroimaging

Antibodies

Antibodies used

Human
1) anti-CD8 (clone SP239, Abcam) diluted at 1:500
2) anti-GRANZYME B (clone GrB-7, Dako) diluted at 1:150

Mouse
3) anti-CD8 (Clone YTS 169.4 Abcam, 1:200)
4) anti-Granzyme B (Polyclonal R&D Systems 1:100)

Validation

References for antibodies
1) Steele KE et al. J Immunother Cancer (2018).
2) Yang et al. JCI Insight (2018)
3) Wang Z et al. Nat Commun (2019).
4) Smyth et al. J. Leukoc. Biol. (1996)

Animals and other organisms

Policy information about studies involving animals; ARRIVE guidelines recommended for reporting animal research

Laboratory animals

All mice were on full C57Bl/6J background.
C57BL/6 (Janvier, France), GzmB-/-, Rag1-/- and OT-I mice (Jackson, United States of America) as CMy-mOva mice (A. Lichtman's lab,

	United States of America)<br>Pigs (Land race White crossed, Lebeau, Gamblais France)
Wild animals	No
Field-collected samples	No
Ethics oversight	Mouse protocol was approved by the ethical committee CEEA34 Université de Paris (APAFIS #10554-2017041016471398).<br>Pig protocol was approved by the ethical committee ComEth Anses/EnvA/UPEC (APAFIS #3841-2016012815406796).

Note that full information on the approval of the study protocol must also be provided in the manuscript.

Human research participants

Policy information about studies involving human research participants

Population characteristics	Characteristics of included patients:<br>sex, age, body mass index, current smoking, family history of coronary disease, history of hypertension, hypercholesterolemia, previous myocardial infarction, previous stroke or transient ischemic attack (TIA), previous cancer, CPK peak, heart failure, renal failure, diabetes, Killip class, left ventricular ejection fraction, STEMI or reperfusion, hospital management (including reperfusion therapy, coronary artery bypass surgery, statins, beta blockers, clopidogrel, diuretics, low molecular weight heparin, GPIIb/IIIa inhibitors)
Recruitment	FAST-MI registry : patients ≥18 years with acute myocardial infarction<br>The time from symptom onset to intensive care unit admission had to be <48 h.
Ethics oversight	The study was approved by the Committee for the Protection of Human Subjects in Biomedical Research of Saint Antoine University Hospital (Ethical Committee) and the data file was declared to the Commission Nationale Informatique et Liberté.

Note that full information on the approval of the study protocol must also be provided in the manuscript.

Clinical data

Policy information about clinical studies

All manuscripts should comply with the ICMJEguidelines for publication of clinical research and a completedCONSORT checklist must be included with all submissions.

Clinical trial registration	ClinicalTrials.gov NCT01237418
Study protocol	Detailled in several published studies. Ex : Puymirat et al. Circulation. 2017 Nov 14;136(20):1908-1919
Data collection	1-month period 2010
Outcomes	1-year mortality

Flow Cytometry

Plots

Confirm that:

- [x] The axis labels state the marker and fluorochrome used (e.g. CD4-FITC).
- [x] The axis scales are clearly visible. Include numbers along axes only for bottom left plot of group (a 'group' is an analysis of identical markers).
- [x] All plots are contour plots with outliers or pseudocolor plots.
- [x] A numerical value for number of cells or percentage (with statistics) is provided.

Methodology

Sample preparation	Cells from the blood, the spleen and ischemic heart tissue
Instrument	FlowJoSoftware (TreeStar)
Software	FlowJoSoftware (TreeStar)
Cell population abundance	No problem of cell population abundance
Gating strategy	Gating strategy was describedin Method Section and in panels (Fig1A, 2A, 2B, S16, S19, S34)

- [x] Tick this box to confirm that a figure exemplifying the gating strategy is provided in the Supplementary Information.
